# Efficacy of eHealth Versus In-Person Cognitive Behavioral Therapy for Insomnia: Systematic Review and Meta-Analysis of Equivalence

**DOI:** 10.2196/58217

**Published:** 2024-08-26

**Authors:** Sofie Møgelberg Knutzen, Dinne Skjærlund Christensen, Patrick Cairns, Malene Flensborg Damholdt, Ali Amidi, Robert Zachariae

**Affiliations:** 1 Department of Psychology and Behavioral Sciences Aarhus University Aarhus Denmark; 2 Department of Oncology Aarhus University Hospital Aarhus Denmark

**Keywords:** sleep disturbance, digital, telehealth, face-to-face, head-to-head comparison, CBTI, cognitive behavioral therapy for insomnia, mobile phone

## Abstract

**Background:**

Insomnia is a prevalent condition with significant health, societal, and economic impacts. Cognitive behavioral therapy for insomnia (CBTI) is recommended as the first-line treatment. With limited accessibility to in-person–delivered CBTI (ipCBTI), electronically delivered eHealth CBTI (eCBTI), ranging from telephone- and videoconference-delivered interventions to fully automated web-based programs and mobile apps, has emerged as an alternative. However, the relative efficacy of eCBTI compared to ipCBTI has not been conclusively determined.

**Objective:**

This study aims to test the comparability of eCBTI and ipCBTI through a systematic review and meta-analysis of equivalence based on randomized controlled trials directly comparing the 2 delivery formats.

**Methods:**

A comprehensive search across multiple databases was conducted, leading to the identification and analysis of 15 unique randomized head-to-head comparisons of ipCBTI and eCBTI. Data on sleep and nonsleep outcomes were extracted and subjected to both conventional meta-analytical methods and equivalence testing based on predetermined equivalence margins derived from previously suggested minimal important differences. Supplementary Bayesian analyses were conducted to determine the strength of the available evidence.

**Results:**

The meta-analysis included 15 studies with a total of 1083 participants. Conventional comparisons generally favored ipCBTI. However, the effect sizes were small, and the 2 delivery formats were statistically significantly equivalent (*P*<.05) for most sleep and nonsleep outcomes. Additional within-group analyses showed that both formats led to statistically significant improvements (*P*<.05) in insomnia severity; sleep quality; and secondary outcomes such as fatigue, anxiety, and depression. Heterogeneity analyses highlighted the role of treatment duration and dropout rates as potential moderators of the differences in treatment efficacy.

**Conclusions:**

eCBTI and ipCBTI were found to be statistically significantly equivalent for treating insomnia for most examined outcomes, indicating eCBTI as a clinically relevant alternative to ipCBTI. This supports the expansion of eCBTI as a viable option to increase accessibility to effective insomnia treatment. Nonetheless, further research is needed to address the limitations noted, including the high risk of bias in some studies and the potential impact of treatment duration and dropout rates on efficacy.

**Trial Registration:**

PROSPERO CRD42023390811; https://www.crd.york.ac.uk/prospero/display_record.php?RecordID=390811

## Introduction

### Background

Insomnia, characterized by difficulties initiating or maintaining sleep, which are perceived as distressing and result in significant impairment of daytime functioning, is a common concern in the general population [1]. It is estimated that approximately 20% of the population experience episodic symptoms of insomnia, resulting in negative consequences for daytime functioning, for example, fatigue, with approximately 10% fulfilling the diagnostic criteria for an insomnia disorder [1]. The association between insomnia and adverse physical and mental health outcomes has been thoroughly documented, with numerous prospective studies showing increased risk of developing cardiovascular disease [2,3], infectious diseases such as the common cold or pneumonia [4,5], all-cause dementia [6,7], mental disorders such as depression and anxiety [8], and social withdrawal and loneliness [9,10]. In addition, not only short but also long sleep duration, both possible indicators of sleep disturbances, have been associated with increased mortality [11,12]. Beyond the personal health implications, insomnia is associated with societal costs through increased health care use, higher levels of work absenteeism, diminished work-related productivity, reduced learning capacity, and poorer academic performance [13-15]. This underlines the extensive societal and economic burdens posed by untreated sleep disturbances.

While hypnotic medications are commonly used to treat insomnia, they are not recommended for long-term use due to the risk of developing tolerance and dependence [16] as well as a wide range of adverse consequences, including daytime drowsiness, impaired cognitive function, increased risk of accidents or falls, and rebound insomnia upon discontinuation [17,18]. Instead, the major sleep medicine and research organizations recommend cognitive behavioral therapy for insomnia (CBTI) as the first-line treatment for insomnia [19-21]. CBTI usually involves a combination of two or more of the following five components [17]: (1) *sleep restriction therapy*, which aims at promoting more efficient and consolidated sleep patterns by first reducing the time spent awake in bed and then gradually allowing the person to increase time in bed [22]; (2) *stimulus-control therapy*, which aims to strengthen the connection between the bed and sleep by associating the bed and bedroom with sleep rather than wakefulness [23]; (3) *relaxation techniques*, which aim to reduce stress, anxiety, and tension that may interfere with falling asleep or staying asleep [24]; (4) *cognitive therapy*, which targets negative thought patterns and maladaptive beliefs about sleep [25]; and (5) *sleep hygiene education*, which focuses on establishing healthy habits and optimizing the sleep environment to promote better sleep [26]. Several meta-analyses have supported the efficacy of CBTI, demonstrating both short-term [16,27] and long-term effects [28] on insomnia, not only as the primary problem but also as a comorbid condition, for example, in patients with chronic pain [29] and survivors of cancer [30]. Compared with pharmacotherapy, CBTI has been found to be at least as effective in reducing insomnia symptoms and generally demonstrates more durable effects [31].

Nonetheless, substantial challenges remain in extending assistance to those affected. Individuals with insomnia rarely receive guideline-compliant treatment, hindered by various obstacles. These include insufficient numbers of trained CBTI providers, low rates of referral by primary care physicians, and geographical and physical barriers that deter patients from receiving help [32-34]. To address these challenges, several alternative eHealth delivery formats of CBTI have been developed and evaluated [32]. These alternatives include telephone- and videoconference-delivered CBTI and fully automated web-based programs and mobile apps.

Recently published meta-analyses have revealed statistically significant and clinically meaningful effects of eHealth CBTI (eCBTI) on various measures, including insomnia severity; self-reported sleep quality; and sleep diary–based outcomes such as sleep onset latency (SOL), wake after sleep onset (WASO), total sleep time (TST), and sleep efficiency [35,36]. This efficacy extends not only to individuals with insomnia as their primary concern [37,38] but also to those with comorbid insomnia, for example, survivors of cancer [39]. However, the results of recent systematic reviews and network meta-analyses comparing various delivery formats of CBTI suggest that in-person–delivered CBTI (ipCBTI) is generally superior to eCBTI, more so for insomnia severity than for sleep diary outcomes [40,41]. In contrast, a network meta-analysis investigating a Food and Drug Administration–authorized prescription eCBTI compared to traditional ipCBTI found that eCBTI was the most efficacious regarding insomnia severity [42].

The inconclusive results of the existing meta-analyses could be due to their reliance on both direct and indirect comparisons and variations in treatment length, dosage, content, and control group types across studies of both formats, which may compromise comparability [41]. To date, no meta-analysis has focused exclusively on randomized controlled trials, conducting direct head-to-head comparisons of eCBTI and ipCBTI, and it thus remains unclear how well the 2 delivery formats compare in terms of efficacy.

In addition, when examining the equivalence or nonequivalence of 2 interventions with meta-analysis, the conventional nonsuperiority null hypothesis test procedure is insufficient. Here, a nonsignificant result merely indicates a failure to reject the null hypothesis of no difference, which cannot conclusively determine nonequivalence or equivalence [43]. To truly test whether treatments are equivalent, we must reject the null hypothesis of nonequivalence, which means that differences in effect sizes (ESs) are as large as or larger than a predetermined equivalence margin [44]. One will usually choose equivalence margins based on previously determined minimal important differences (MIDs), referring to the minimal difference in an outcome of interest that can be viewed as clinically meaningful [45].

### Objectives

Given the prevalence of insomnia and the need for diverse treatment approaches, establishing the equivalence or nonequivalence of digital and traditional CBTI delivery formats is crucial. The possible equivalence or nonequivalence of eCBTI and ipCBTI has not yet been subjected to meta-analysis. The aim of this study was, therefore, to test the comparability of eCBTI and ipCBTI with a systematic review and meta-analysis of equivalence based on randomized controlled trials directly comparing the 2 delivery formats.

## Methods

This study was registered with PROSPERO (registration CRD42023390811) and conducted in accordance with the PRISMA (Preferred Reporting Items for Systematic Reviews and Meta-Analysis) statement (Multimedia Appendix 1) [46].

### Search Strategy

The electronic databases of CINAHL, Cochrane, Embase, PsycINFO, and PubMed were searched for publications from the earliest time available until January 5, 2024. Keywords related to insomnia (eg, sleep disturbance OR sleep disorder) were combined with keywords related to CBTI (eg, cognitive behavioral OR CBT) and keywords pertaining to eHealth (eg, telehealth OR digital). The search strings were constructed in collaboration with a skilled librarian. Table S1 in Multimedia Appendix 2 comprises detailed search strings for each database. The electronic database searches were supplemented with backward searches of reference lists of included studies. No separate protocol in addition to the one registered with PROSPERO was prepared. The main methodological changes to the original registered protocol were as follows: (1) the search date was changed from 1991 to the earliest time available due to the inclusion of additional electronic delivery formats, for example, telephone-based interventions, and (2) we also extracted data on secondary nonsleep outcomes of fatigue, anxiety, and depression.

### Inclusion and Exclusion Criteria

On the basis of the Population, Intervention, Comparison, and Outcome (PICO) approach [47], the inclusion criteria in Textbox 1 were used.

**Inclusion criteria.**

**Population**

- Adults and adolescents (aged ≥12 years) with (1) self-reported poor sleep quality or symptoms of insomnia assessed with relevant instruments, for example, the Insomnia Severity Index (ISI) [48] or the Pittsburgh Sleep Quality Index (PSQI) [49], or (2) an insomnia diagnosis established by a structured clinical interview. Studies of children aged <12 years and studies focusing on other medical sleep disorders (eg, sleep apnea and narcolepsy) were excluded. No exclusions were made based on comorbid disorders.

**Intervention**

- Electronically delivered eHealth cognitive behavioral therapy for insomnia (eCBTI), defined as cognitive behavioral therapy for insomnia (CBTI) delivered remotely or using digital means without in-person contact, for example, CBTI delivered via telephone or video, web-based CBTI, or smartphone-based CBTI. CBTI was defined as any combination of ≥2 of the standard CBTI components, that is, sleep restriction therapy, stimulus-control therapy, relaxation, cognitive therapy, and sleep hygiene education. Other eHealth interventions aimed at treating insomnia, for example, mindfulness-based interventions, were excluded, as were stand-alone CBTI components.

**Comparison**

- eCBTI had to be directly compared with in-person–delivered CBTI (ipCBTI), defined as any combination of ≥2 standard CBTI components delivered in person, either individually or in a group format. Other in-person–delivered interventions aimed at treating insomnia, including stand-alone CBTI components, were excluded.

**Outcomes**

- Studies should report means with SDs or SEs; change scores; effect sizes (eg, Cohen *d*) or data that could be converted into an effect size for at least 1 relevant sleep outcome, that is, insomnia severity or clinically significant sleep disturbance assessed with relevant scales such as the ISI [48] and the PSQI [49]; structured clinical interviews; or a relevant sleep parameter assessed with a sleep diary, actigraphy, or polysomnography. Only randomized controlled trials published in English in peer-reviewed journals were included. Case studies, open trials, and other nonrandomized controlled trials were excluded, together with studies with sample sizes <10.

### Study Selection and Data Extraction

Identified references were imported into the web-based software program Covidence (Veritas Health Innovation) [50]. After duplicate removal, title and abstract screening was performed, followed by full-text screening. One author (SMK) conducted the final search, with 3 authors (SMK, DSC, and PC) conducting the screening process independently. Discrepancies were resolved through discussions and, in case of disagreement, by including a fourth author (RZ). The primary outcome was total sleep disturbance calculated as the combined, that is, averaged, results for insomnia severity and sleep quality assessed with validated scales, for example, the Insomnia Severity Index (ISI), the Pittsburgh Sleep Quality Index (PSQI), or similar scales. Secondary sleep outcomes were insomnia severity measured with the ISI; sleep quality measured with the PSQI; and the sleep diary or actigraphy-based outcomes of SOL, WASO, TST, and sleep efficiency calculated as TST relative to time in bed. In addition, we extracted data on the secondary nonsleep outcomes of fatigue, anxiety, and depression, as well as for study characteristics that could potentially explain (moderate) any variations in the differences between ipCBTI and eCBTI, including mean sample age, the proportion of women in the sample, study dropout rates, the type and degree of therapist contact, the number of treatment sessions, treatment length, and the type and number of CBTI components in each condition. A total of 3 authors (SMK, DSC, and PC) extracted data from the included studies independently, and discrepancies were resolved through discussion and by including a fourth author (RZ).

### Risk of Bias Assessment

The revised Cochrane Risk of Bias tool [51] was used to evaluate the risk of bias in the included studies. Five sources of bias were assessed: (1) bias arising from the randomization process, (2) bias due to deviations from intended interventions, (3) bias due to missing outcome data, (4) bias in the measurement of the outcome, and (5) bias in the selection of the reported result. All studies were evaluated for each of the 5 potential sources of bias and rated as either low risk, high risk, or some concerns on the primary outcome of sleep disturbance. In addition, an overall assessment of the risk of bias was conducted for each study. As the number of dropouts in studies investigating eCBTI is generally high, with mean attrition rates ranging from 22% to 25% [36,52], it was decided to use a less conservative criterion in domain 3. We thus considered the availability of data from ≥90% of the participants at the postintervention assessment time-point sufficient. The assessments were conducted independently by 3 authors (SMK, DSC, and PC). Disagreements were solved by negotiation.

### Data Analysis

Hedges' *g*, a variation of Cohen *d* correcting for a possible bias due to a small sample size [53], was used as the standardized ES. All ES calculations were based on differences between the ipCBTI and eCBTI intervention groups in changes (means and SDs) from preintervention to postintervention time points and from preintervention to follow-up time points, standardized by change score SDs. If the relevant data were not reported, we contacted the authors, requesting them to provide this information. We also analyzed the mean differences across the different sleep-related outcomes, that is, mean differences in ISI and PSQI scores; percentages for sleep efficiency; and minutes for SOL, WASO, and TST. ESs were pooled using the inverse variance method, taking the precision of each study into account. A random-effects model was used in all analyses, with positive ESs indicating ipCBTI being more efficacious than eCBTI. If studies reported results for >1 measure per outcome, for example, insomnia severity or sleep quality, we chose the most commonly used outcome measure, that is, the ISI for insomnia severity and the PSQI for sleep quality, so that only 1 result per study was used in each data synthesis, ensuring the independence of results.

Differences between ipCBTI and eCBTI were first analyzed using a conventional random-effects test of superiority for results at both postintervention and follow-up. The pooled ESs were then subjected to analyses of equivalence [44], testing whether the CI fell within an equivalence interval based on the clinical significance thresholds (or MIDs) previously suggested for the various sleep outcome measures. Thus, the MIDs were 0.5 SD for the standardized mean differences of sleep disturbance, insomnia severity, sleep quality, sleep efficiency, SOL, WASO, and TST, as suggested in a previous study [54]. The MIDs for the mean differences were 4.4 points on the PSQI [55], 5% for sleep efficiency, 10 minutes for SOL, and 15 minutes for WASO and TST [54]. The 6-point MID previously suggested for the ISI [56] was based on an analysis of within-subject improvement, that is, minimal important change. We therefore used 0.5 × the average SD of 4.2 (2.6 points) for ISI at baseline in patients with insomnia reported in the original validation paper [48]. This SD corresponds well with the average SD of 4.1 found for ISI scores across studies at baseline in this review. The equivalence interval of SD 0.25 for depression was chosen based on the MID previously suggested [57]. As no specific MIDs were available for the measures of fatigue and anxiety, 0.5 SD was chosen as the equivalence interval for these measures [58]. The equivalence test is based on two 1-sided tests, with the 2 interventions considered to be statistically significantly equivalent if the largest of the 2 *P* values is <.05 [44].

Heterogeneity was explored by calculating the *I*^2^ statistic [59,60]. In addition, we calculated the 95% prediction interval, that is, the interval in which 95% of future observations from the same family of studies are expected to fall [61]. Possible reasons for heterogeneity of the differences between ipCBTI and eCBTI were explored with moderator analyses comparing the ESs of studies according to the following study characteristics: mean sample age, the proportion of women in the sample (%), overall study dropout (%), the difference in dropout between eCBTI and ipCBTI (%), therapist contact in the eCBTI condition (reference: fully automated, ie, no direct or indirect therapist contact), the number of treatment sessions, and treatment duration (weeks). In addition, we explored the possible role of the number of CBTI components used in ipCBTI and eCBTI, respectively. Both categorical and continuous moderators were analyzed with meta-regression when K (the number of studies in the analysis) was ≥10.

When K was ≥10, the possibility of publication bias was evaluated with funnel plots and the method developed by Egger et al [62]. If the results were suggestive of publication bias, we planned to calculate an adjusted ES using the Duval and Tweedie trim and fill method [63]. The calculations were conducted with Comprehensive Meta-Analysis (version 4; Biostat, Inc) [64] and various formulas in Microsoft Excel.

Finally, to assess the potential efficacy of each condition, we calculated the pooled within-group differences for each condition at postintervention and follow-up for all outcomes.

### Supplementary Bayesian Analyses

To aid the interpretation of the results, we conducted a supplementary Bayesian model-averaged meta-analysis [65] of the overall comparisons of ipCBTI and eCBTI at postintervention and follow-up. The procedure examined the results of four models: (1) the fixed-effect null hypothesis, that is, the difference between ipCBTI and eCBTI is nonzero (fH_0_), (2) the fixed-effect alternative hypothesis, that is, the difference is zero (fH_1_), (3) the random-effects null hypothesis (rH_0_), and (4) the random-effects alternative hypothesis (rH_1_). The Bayesian model-averaged analysis thus avoids selecting either a fixed- or random-effects model and addresses 2 questions in light of the observed data: What is the plausibility that the overall effect is 0, that is, equivalent? and Is there a between-study variability in the ESs? Bayesian methods enable direct probability statements about the hypotheses themselves and avoid other issues associated with null hypothesis significance testing, such as the overreliance on relatively arbitrary *P* value thresholds and the dichotomization of results into “significant” and “nonsignificant” [66]. We chose an uninformed prior probability, that is, 25%, of the 4 models and 2000 iterations. Concerning parameter distributions, we chose previously recommended defaults [65], using a 0-centered Cauchy prior with a scale of 0.707 for the ES and an empirically informed prior distribution of nonzero between-study deviation estimates from 705 meta-analyses [67]. This distribution has been approximated by an inverse-gamma (1, 0.15) prior on the SD (Tau) [65]. The Bayesian analyses were conducted with the computer software JASP (version 16; University of Amsterdam) [68]. All data included in this review are available in tables and figures in the manuscript or Multimedia Appendix 2.

## Results

### Study Selection

**Figure 1:**
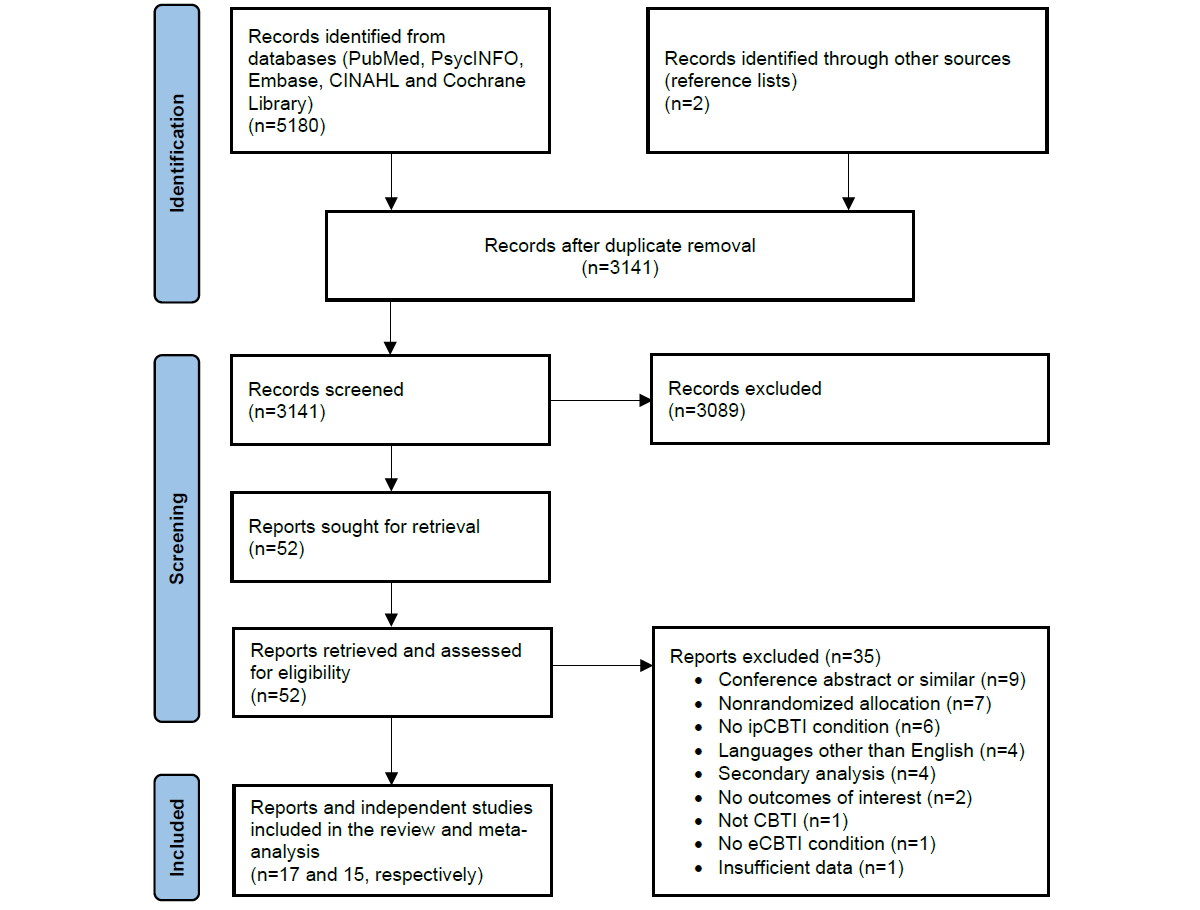
Study selection flowchart. CBTI: cognitive behavioral therapy for insomnia; eCBTI: eHealth cognitive behavioral therapy for insomnia; ipCBTI: in-person–delivered cognitive behavioral therapy for insomnia.

A total of 5180 records were identified via databases, and 2 additional records were identified via reference lists. After 2039 (39.36%) duplicates were removed, 3141 (60.64%) references were screened by title and abstract. Full-text screening was carried out for 1.66% (52/3141) records, and after assessing eligibility, we identified 32% (17/52) full-text reports of 15 unique randomized head-to-head comparisons of ipCBTI and eCBTI. The results of the study selection process are shown in Figure 1. A list of studies excluded after full text screening with reasons for exclusion is provided in Table S2 in Multimedia Appendix 2.

### Study Characteristics

The characteristics of the included studies are summarized in Table 1. Of the 15 studies, most studies were conducted in the United States (K=5, 33%), followed by Canada (K=3, 20%) and the Netherlands (K=2, 13%). The rest of the studies (K=5, 33%) were all conducted in different countries. A total of 1083 participants were included in the 15 studies and randomized to eCBTI and ipCBTI treatment conditions. A little more than half, 666/1083 (61.5%), were women. The mean age of the total sample was 40.6 (SD 11.3) years, with mean sample ages ranging from 15.5 to 55.1 years. Of the 15 studies, most focused on insomnia as the primary problem (K=11, 73%), and 4 studies (27%) focused on patients with comorbid insomnia, that is, people maintaining sobriety from alcohol (K=1, 25%), survivors of breast cancer (K=1, 25%), and patients with posttraumatic stress disorder (K=2, 50%). All studies had insomnia as an inclusion criterion, based on diagnostic criteria; validated questionnaires; or quantitative criteria such as SOL, WASO, or early morning awakenings of ≥30 minutes occurring at least 3 nights a week, or sleep efficiency <85%.

**Table 1:** Study characteristics.

Study, year	Country	Participant characteristics, insomnia type, and sample type	Demographic characteristics: mean age, (SD); women, n/N (%)	eCBTI^a^ and ipCBTI^b^ participants, n/N (%)	eCBTI treatment format, delivery mode, therapist contact, and the number of sessions	ipCBTI treatment format and the number of sessions	Sleep outcomes	Secondary outcomes	Time to postintervention and time to follow-up (weeks)	eCBTI and ipCBTI study dropout (%)^c^; treatment dropout (%)^d^	eCBTI and ipCBTI components, n	ROB-2^e^ (low risk, some concerns, or high risk)
Currie et al [69], 2004	Canada	Adults, comorbid insomnia (people maintaining sobriety from alcohol), and clinical sample	43.3 (10.9); 18/60 (30)	28/57 (49) and 29/57 (51)	Individual, self-help and telephone, synchronous, and 5 sessions	Individual and 5 sessions	Insomnia severity, SQ^f^, and sleep diary (SOL^g^, WASO^h^, TST^i^, and sleep efficiency)	Depression	0 and 26	50% and 35%; NR^j^ and NR	5 and 5	Some concerns
Bastien et al [70], 2004	Canada	Adults, primary insomnia, and community sample	42.8 (9.7); 18/29 (62)	14/29 (48) and 15/29 (52)	Individual, telephone, synchronous, and 8 sessions	Individual and 8 sessions	Insomnia severity and sleep diary (SOL, WASO, TST, TIB^k^, and sleep efficiency)	Anxiety and depression	0 and 26	36% and 53%; 0% and 0%	4 and 4	High risk
Savard et al [71], 2014, and Savard et al [72], 2016	Canada	Adults, comorbid insomnia (breast cancer), and clinical sample	53.9, (8.8); 161/161 (100)	80/161 (49.7) and 81/161 (50.3)	Individual, video-based self-help with phone support, synchronous, and 6 sessions	Individual and 6 sessions	Insomnia severity and sleep diary (SOL, WASO, TST, and sleep efficiency)	Fatigue, anxiety, and depression	0 and 52	39% and 26%; NR and NR	4 and 4	High risk
Blom et al [73], 2015	Sweden	Adults, primary insomnia, and community sample	54.4 (13.8); 23/48 (48)	24/48 (50) and 24/48 (50)	Individual, web based with written feedback, asynchronous, and 8 sessions	Group and 8 sessions	Insomnia severity and sleep diary (SOL, TST, and sleep efficiency)	Depression	0 and 26	46% and 46%; 29% and 17%	5 and 5	Some concerns
de Bruin et al [74], 2015, and de Bruin et al [75], 2018	The Netherlands	Adolescents, primary insomnia, and community sample	15.4 (1.6); 59/77 (77)	39/77 (51) and 38/77 (49)	Individual, web based with written feedback and chat, mixed, and 6 sessions	Group and 6 sessions	Insomnia severity and sleep diary and actigraphy (SOL, WASO, TST, TIB, and sleep efficiency)	Anxiety and depression	0 and 9-52	18% and 5%; 0 and 0	5 and 5	Some concerns
Lancee et al [76], 2016	The Netherlands	Adults, primary insomnia, and community sample	39.9 (13.6); 48/60 (80)	30/60 (50) and 30/60 (50)	Individual, web based with email feedback, asynchronous, and 6 sessions	Individual and 6 sessions	Insomnia severity and sleep diary (TST and sleep efficiency)	Anxiety and depression	4 and 26	30% and 13%; 23% and 7%	5 and 5	High risk
Taylor et al [77], 2017	United States	Adults, primary insomnia, and sample of army personnel	32.7 (7.4); 13/67 (19)	34/67 (51) and 33/67 (49)	Individual, web based (automated), none, and 6 sessions	Individual and 6 sessions	Insomnia severity and sleep diary and actigraphy (SOL, WASO, TST, and sleep efficiency)	—^l^	0 and N/A^m^	41% and 58%; 21% and 12%	5 and 5	High risk
Laurel Franklin et al [78], 2017	United States	Adults, comorbid insomnia (PTSD^n^), and clinical sample	53.8 (12.0); 0/18 (0)	11/18 (61) and 7/18 (39)	Individual, telephone, synchronous, and 6 sessions	Individual and 6 sessions	SQ	—	0 and 13	46% and 14%; 18% and 0%	5 and 5	High risk
Gieselmann and Pietrowsky [79], 2019	Germany	Adults, primary insomnia, and community sample	39.5, (13.1); 26/50 (52)	23/50 (46) and 27/50 (54)	Individual, chat, synchronous, and 3 sessions	Individual and 3 sessions	SQ and sleep diary and actigraphy (SOL, TST, and sleep efficiency)	Fatigue, anxiety, and depression	0 and 9	4% and 34%; NR and NR	2 and 2	Some concerns
Gehrman et al [80], 2020	United States	Adults, comorbid insomnia (PTSD), and clinical sample	55.1 (12.2); 9/95 (10)	49/96 (51) and 47/96 (49)	Group, video conferencing, synchronous, and 6 sessions	Group and 6 sessions	Insomnia severity and SQ	—	2 and 13	63% and 47%; 29% and 26%	5 and 5	Some concerns
Arnedt et al [81], 2021	United States	Adults, primary insomnia, and Community sample	47.2 (16.3); 46/65 (71)	33/65 (51) and 32/65 (49)	Individual, video conferencing, synchronous, and 6 sessions	Individual and 6 sessions	Insomnia severity and sleep diary (SOL, WASO, TST, and sleep efficiency)	Fatigue, anxiety, and depression	0 and 13	NR and NR; 6% and 3%	5 and 5	Some concerns
Gehrman et al [82], 2021	United States	Adults, primary insomnia, and community sample	33.4 (10.3); 26/41 (63)	21/41 (51) and 20/41 (49)	Individual, video conferencing, synchronous, and 8 sessions	Individual and 8 sessions	Insomnia severity	Fatigue, anxiety, and depression	3 and 13	24% and 30%; 19% and 30%	5 and 5	Some concerns
Kallestad et al [83], 2021	Norway	Adults, primary insomnia, and clinical sample	41.3 (11.6); 76/101 (75.2)	49/101 (48.5) and 52/101 (51.5)	Individual, web based (automated), none, and 6 sessions	Individual and 8 sessions	Insomnia severity and sleep diary (SOL, WASO, TST, and sleep efficiency)	Fatigue	0 and 26	16% and 8%; 12% and 0%	4 and 4	Some concerns
Wong et al [84], 2021	Hong Kong	Adolescents or older participants, primary insomnia, and community sample	37.6 (15.3); 90/140 (64.3)	70/140 (50) and 70/140 (50)	Individual, web-based self-help, none, and 4 sessions	Group and 1 session (workshop)	Insomnia severity	Anxiety and depression	4 and 12	26% and 30%; NR and 49%	5 and 5	Some concerns
Chan et al [85], 2022	China	Youth, primary insomnia, and community sample	20.2 (2.4); 61/90 (69)	45/90 (50) and 45/90 (50)	Individual, email self-help, none, and 8 sessions	Group, and 8 sessions	Insomnia severity, SQ, and sleep diary (SOL, WASO, TST, TIB, and sleep efficiency)	Fatigue, anxiety, and depression	1 and 26	47% and 22%; 38% and 4%	5 and 4	High risk

^a^eCBTI: eHealth cognitive behavioral therapy for insomnia.

^b^ipCBTI: in-person–delivered cognitive behavioral therapy for insomnia.

^c^Study dropout: proportion of participants lost to follow-up at the most distant time point after baseline.

^d^Treatment dropout: proportion of participants who dropped out of treatment (defined as completing <50% of treatment cores or sessions).

^e^ROB 2: Cochrane Risk of Bias.

^f^SQ: sleep quality.

^g^SOL: sleep onset latency.

^h^WASO: wake after sleep onset.

^i^TST: total sleep time.

^j^NR: not reported.

^k^TIB: time in bed.

^l^No data.

^m^N/A: not applicable.

^n^PTSD: posttraumatic stress disorder.

Sleep outcomes reported in the 15 included studies were insomnia severity (K=13, 87%), with the ISI being the most frequently used instrument (K=11, 85%), and sleep quality (K=5, 33%), with the PSQI being used by all studies reporting this outcome. Sleep diaries and actigraphy were used in 11 (73%) and 3 (20%) studies, respectively, assessing sleep parameters such as SOL, WASO, TST, time in bed, and sleep efficiency. A total of 11 (73%) studies assessed depression, with the Hospital Anxiety and Depression Scale (HADS) [86] (K=3, 27%) being the most frequently used, followed by the Beck Depression Inventory (BDI) [87] (K=2, 18%), the Patient Health Questionnaire (PHQ) [88] (K=2, 18%), and the Center for Epidemiologic Studies Depression Scale (CES-D) [89] (K=2, 18%). Of the 15 studies, 9 (60%) studies assessed anxiety, with most (K=4, 44%) using the HADS, followed by the General Anxiety Disorder-7 (GAD-7) [90] (K=2, 22%), and 7 (47%) studies assessed fatigue, with most frequently using the Multidimensional Fatigue Inventory (MFI) [91] (K=4, 57%). A total of 14 studies reported follow-up data, with time to follow-up ranging from 9 to 52 weeks.

In all but 1 (7%) of the 15 studies, eCBTI was delivered individually. In most studies (K=10, 67%), eCBTI involved some degree of interaction with a treatment provider, with real-time, synchronous therapist contact being available in 8 (53%) studies and asynchronous support, for example, via email, being offered in 2 (13%) studies. One (7%) study provided both synchronous and asynchronous therapist contact. In 4 (27%) studies, eCBTI was provided completely without interaction with a treatment provider, for example, in a fully automated format. ipCBTI was primarily delivered individually (K=10, 67%), with 5 (33%) studies using a group format.

### Risk of Bias

The risk of bias in the individual studies is summarized in Figure S1 in Multimedia Appendix 2. Of the 15 studies, 10 (67%) were characterized as having some concerns regarding the overall risk of bias, and the remaining 5 (33%) were classified as having a high risk of bias overall. No studies were characterized as having a low risk of bias. The reasons for a study being categorized as having a high risk of bias stemmed primarily from “bias due to missing outcome data” due to the combination of high rates of missing outcome data and failure to include analyses correcting for this, for example, sensitivity analyses. Bias raising “some concerns” primarily stemmed from “bias in the measurement of the outcome” due to the combination of using a self-reported outcome and nonblinding. Only few studies had attempted some element of blinding. Of the 15 studies, 3 (20%) [79,81,84] reported that participants were kept blind to study hypotheses, 1 (7%) reported that treatment providers were kept blind to study hypotheses [79], and 2 (13%) reported that the data analyst was blinded to the allocation status of the participants [83,84]. In addition, bias raising “some concerns” stemmed from “bias in the selection of the reported result” due to inadequate preregistration of the analytical strategy.

### Comparing Intervention Characteristics of ipCBTI and eCBTI

More participants had dropped out of eCBTI than out of ipCBTI at both postintervention (104/528, 19.7% vs 72/526, 13.7%) and follow-up (176/509, 34.6% vs 145/509, 28.5%). However, the differences did not reach statistical significance (*P*=.17 and .46). No between-condition differences were found in the mean number of sessions (6.0 vs 6.1; *P*=.91), the duration of the intervention (6.7 weeks vs 6.6 weeks; *P*=.83), or the number of CBTI components (4.5 vs 4.6; *P*=.83).

### Within-Group Effects

As presented in Table S3 in Multimedia Appendix 2, statistically significant (*P*<.05) improvements from preintervention to postintervention time points and from preintervention to follow-up time points were observed for both ipCBTI and eCBTI for all self-reported sleep outcomes. At postintervention time points, the ESs (Hedges' *g*) ranged from 0.27 (TST) to 1.97 (ISI) for ipCBTI and from 0.23 (TST) to 1.36 (ISI) for eCBTI. Similarly, at follow-up, the ESs ranged from 0.43 (TST) to 1.88 (ISI) for ipCBTI and from 0.39 (TST) to 1.41 (total sleep disturbance) for eCBTI. For the few actigraphy-based sleep outcomes at postintervention (K=3, 20%), only the results for SOL in the ipCBTI condition (Hedges' *g*=0.53; mean difference=–11.5 minutes) reached statistical significance. At postintervention time points, in the ipCBTI condition, the ISI total score was, on average, improved by 9.0 points; the PSQI global score by 4.4 points; diary-based sleep efficiency by 12.1%; and diary-based SOL, WASO, and TST by –20.9, –23.5, and +21.3 minutes, respectively. The comparable results for eCBTI were 7.1 points, 3.5 points, 10.3%, –19.6 minutes, –19.5 minutes, and +16.3 minutes, respectively. As also seen in Table S3 in Multimedia Appendix 2, the within-participant improvements in fatigue, anxiety, and depression were all statistically significant and similar for both delivery formats at postintervention. Similar results were found for the secondary nonsleep outcomes at follow-up (data not shown).

### Conventional Superiority Meta-Analysis

**Figure 2:**
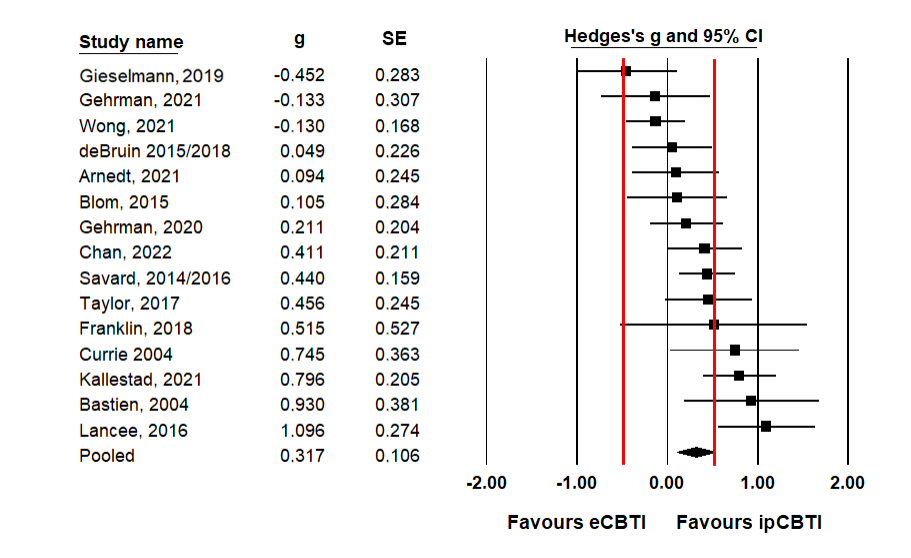
Forest plot of postintervention differences (Hedges' *g*) between the effects of eHealth cognitive behavioral therapy for insomnia (eCBTI) and in-person–delivered cognitive behavioral therapy for insomnia (ipCBTI) on total sleep disturbance (red lines denote the equivalence margin).

**Figure 3:**
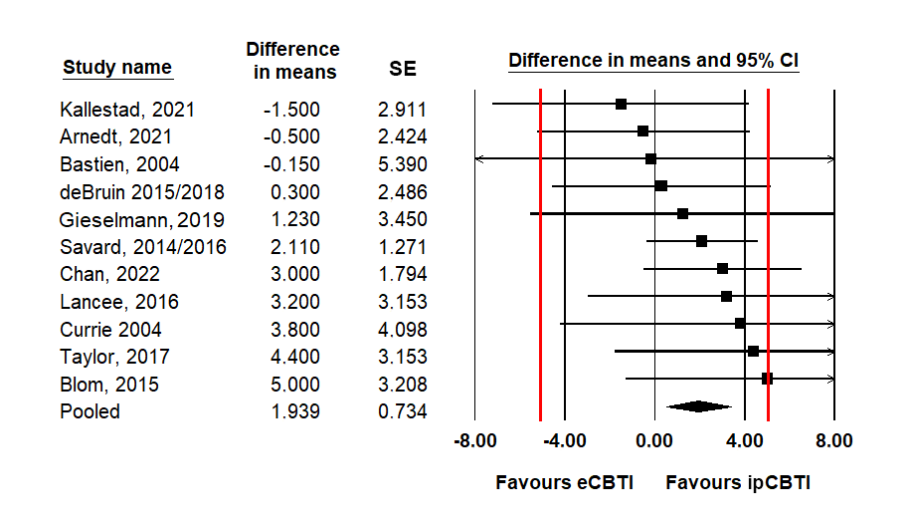
Forest plot of postintervention mean differences (%) between the effects of eHealth cognitive behavioral therapy for insomnia (eCBTI) and in-person–delivered cognitive behavioral therapy for insomnia (ipCBTI) on sleep efficiency (red lines denote the equivalence margin).

**Figure 4:**
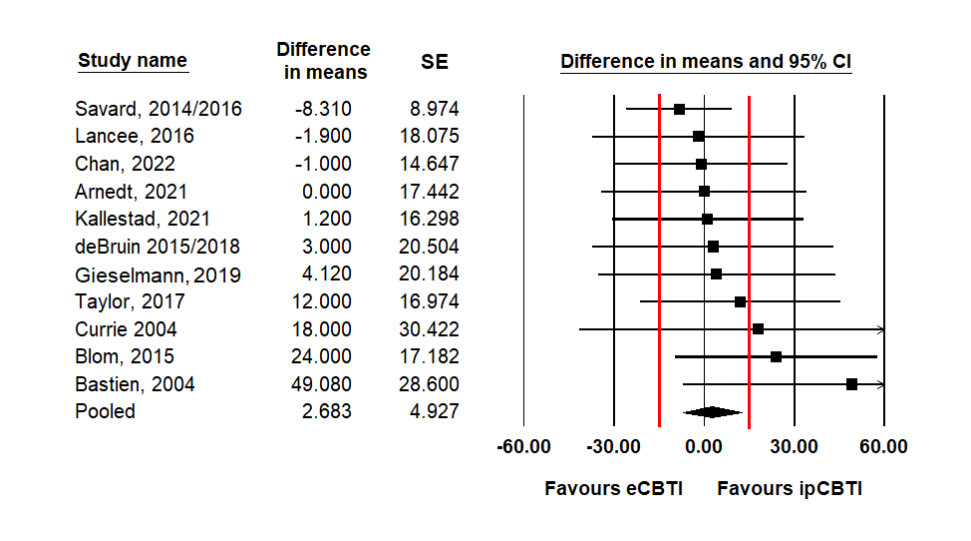
Forest plot of postintervention mean differences (min) between the effects of eHealth cognitive behavioral therapy for insomnia (eCBT) and in-person–delivered cognitive behavioral therapy for insomnia (ipCBTI) on total sleep time (red lines denote the equivalence margin).

As presented in Table 2, when analyzed with conventional superiority meta-analysis, the pooled differences between ipCBTI and eCBTI reached statistical significance in 11 (34%) out of 32 comparisons. The effects generally favored ipCBTI but were small, for example, corresponding to a mean difference of 1.8 points on the ISI and 1.9% in sleep efficiency. The pooled difference for total sleep disturbance corresponded to a small ES (Hedges' *g*=0.32). The forest plots are shown in Figures 2-4 [69-85] and Figures S2-S11 in Multimedia Appendix 2. Concerning the secondary nonsleep outcomes of fatigue, anxiety, and depression, no differences in the conventional superiority analyses reached statistical significance (Table S4 in Multimedia Appendix 2).

**Table 2:** Results of a meta-analysis of studies directly comparing the efficacy of in-person–delivered cognitive behavioral therapy for insomnia (ipCBTI) and digitally delivered eHealth CBTI (eCBTI), including tests of nonzero differences and tests of statistical equivalence.

ipCBTI vs eCBTI					Heterogeneity			Pooled effect				Equivalence^a^	
Outcome		K^b^	N^c^	*I* ^2^		*T* ^2^	Effect^d^ (95% CI)		*P* value^e^	95% PI^f,g^	MID^h^		*P* value
**Self-reported sleep outcomes at postintervention**													
	Total sleep disturbance (Hedges' *g*)	15	1068	63.5		0.10	0.32 (0.11 to 0.53)		.003^i^	–0.41 to 1.04	0.5 SD^j^		.04^i^
	ISI^k^, mean difference (points)	11	897	70.0		2.35	–1.8 (–2.9 to –0.7)		.002^i^	–5.48 to 1.92	2.6^l^		.07
	ISI (Hedges' *g*)	11	897	63.7		0.09	0.37 (0.15 to 0.60)		.001^i^	–0.35 to 1.10	0.5 SD^j^		0.13
	PSQI^m^, mean difference (points)	5	279	63.1		1.48	–0.9 (–2.3 to 0.6)		.23	–5.35 to 3.64	4.4^n^		<.001^i^
	PSQI (Hedges' *g*)	5	279	62.0		0.13	0.26 (–0.15 to 0.67)		.22	–1.06 to 1.58	0.5 SD^j^		.12
	Sleep efficiency^o^ diary^p^, mean difference (%)	11	779	00.0		0.00	1.9 (0.5 to 3.4)		.01^i^	—^q^	5%^r^		<.001^i^
	Sleep efficiency diary (Hedges' *g*)	11	779	00.0		0.00	0.17 (0.03 to 0.31)		.02^i^	—	0.5 SD^j^		<.001^i^
	SOL^s^ diary, mean difference (min)	10	719	00.0		0.00	–2.6 (–6.5 to 1.2)		.18	—	10 min^t^		<.001^i^
	SOL diary (Hedges' *g*)	10	719	2.0		0.00	0.07 (–0.08 to 0.21)		.39	–0.13 to 0.26	0.5 SD		<.001^i^
	WASO^u^ diary, mean difference (min)	8	621	14.7		7.80	–2.5 (–7.5 to 2.6)		.34	–11.79 to 6.82	15 min^v^		<.001^i^
	WASO (Hedges' *g*)	8	621	12.7		0.01	0.09 (–0.08 to 0.26)		.30	–0.21 to 0.39	0.5 SD^w^		<.001^i^
	TST^x^, mean difference (min)	11	779	00.0		0.00	–2.7 (–12.3 to 7.0)		.59	—	15 min^y^		.01^i^
	TST (Hedges' *g*)	11	779	00.0		0.00	0.05 (–0.09 to 0.19)		.52	—	0.5 SD^w^		<.001^i^
**Self-reported sleep outcomes at follow-up**													
	Total sleep disturbance (Hedges' *g*)	14	988	58.1		0.08	0.24 (0.04 to 0.45)		.02^i^	–0.42 to 0.90	0.5 SD^j^		.01^i^
	ISI, mean difference (points)	10	817	65.7		1.98	–1.3 (–2.4 to –0.2)		.02^i^	–4.81 to 2.20	2.6^l^		<.001^i^
	ISI (Hedges' *g*)	10	817	61.4		0.08	0.27 (0.04 to 0.50)		.02^i^	–0.44 to 0.98	0.5 SD^j^		.03^i^
	PSQI, mean difference (points)	5	279	55.8		1.20	–0.8 (–2.1 to 0.6)		.28	–4.87 to 3.37	4.4^n^		<.001^i^
	PSQI (Hedges' *g*)	5	279	53.8		0.09	0.21 (–0.16 to 0.58)		.27	–0.92 to 1.34	0.5 SD^j^		.06
	Sleep efficiency diary, mean difference (%)	10	700	52.0		7.81	2.8 (0.3 to 5.4)		.03^i^	–4.26 to 9.92	5%^r^		.047^i^
	Sleep efficiency diary (Hedges' *g*)	10	700	51.3		0.06	0.25 (0.03 to 0.47)		.03^i^	–0.38 to 0.88	0.5 SD^j^		.01^i^
	SOL diary, mean difference (min)	9	640	9.0		4.46	–4.8 (–9.3 to –0.2)		.04^i^	–12.14 to 2.65	10 min^t^		.01^i^
	SOL diary (Hedges' *g*)	9	640	22.0		0.02	0.14 (–0.04 to 0.32)		.12	–0.23 to 0.52	0.5 SD		<.001^i^
	WASO diary, mean difference (min)	7	542	37.1		24.2	0.6 (–5.9 to 7.0)		.87	–14.66 to 15.77	15 min^v^		<.001^i^
	WASO diary (Hedges' *g*)	7	542	35.4		0.03	–0.02 (–0.24 to 0.20)		.86	–0.55 to 0.51	0.5 SD^w^		<.001^i^
	TST, mean difference (min)	10	700	38.3		193.2	6.3 (–8.2 to 20.9)		.39	–29.98 to 42.66	15 min^y^		.12
	TST (Hedges' *g*)	10	700	36.9		0.03	0.08 (–0.11 to 0.28)		.40	–0.40 to 0.57	0.5 SD^w^		<.001^i^
**Actigraphy-based sleep outcomes at postintervention**													
	Sleep efficiency actigraphy, mean difference (%)	3	194	00.0		0.00	–0.8 (–2.9 to 1.3)		.47	—	5%^r^		<.001^i^
	Sleep efficiency actigraphy (Hedges' *g*)	3	194	00.0		0.00	–0.09 (–0.37 to 0.19)		.53	–1.91 to 1.73	0.5 SD^j^		.002^i^
	SOL actigraphy, mean difference (min)	3	194	00.0		0.00	–2.6 (–8.9 to 3.6)		.41	–43.07 to 37.83	10 min^t^		.01^i^
	SOL actigraphy (Hedges' *g*)	3	194	00.0		0.00	0.14 (–0.14 to 0.42)		.32	–1.68 to 1.96	0.5 SD^j^		.01^i^
	TST actigraphy, mean difference (min)	3	194	2.8		7.04	–16.9 (–34.6 to 0.8)		.06	–136.2 to 102.4	15 min^y^		.42
	TST actigraphy (Hedges' *g*)	3	194	31.2		0.03	–0.24 (–0.58 to 0.10)		.17	–3.33 to 2.87	0.5 SD^j^		.09

^a^The test of equivalence tests whether the CI falls within an equivalence interval. The equivalence test is based on the largest *P* value from two 1-sided tests [44].

^b^K: number of studies.

^c^N: total number of participants.

^d^Analyses were conducted for outcomes with K≥3 for mean differences (%, min) and standardized mean difference (SMD; adjusted for small sample bias; Hedges' *g*) [53], with positive values of Hedges' *g* indicating a difference of effects in favor of ipCBTI compared with eCBTI.

^e^2-tailed *P* values.

^f^PI: prediction interval.

^g^95% prediction interval is the interval in which 95% of future observations from the same family of studies will fall [61].

^h^MID: minimal important difference (or clinical significance threshold) [54].

^i^Statistically significant *P* values (*P*<.05) indicate equivalence.

^j^SMD=0.50, as suggested by Edinger et al [54].

^k^ISI: Insomnia Severity Index.

^l^2.6 point difference on the Insomnia Severity Index (ISI), corresponding to 0.5 × SD found in the original validation study (SD 4.2) [48] (average ISI baseline SD across studies in this review=4.1).

^m^PSQI: Pittsburgh Sleep Quality Index.

^n^4.4 point difference on the Pittsburgh Sleep Quality Index (PSQI), as suggested by Longo et al [55].

^o^Sleep efficiency (%; total sleep time/time in bed × 100).

^p^Diary: sleep parameters based on sleep diaries, for example, the Consensus Sleep Diary [92].

^q^Not applicable.

^r^5% difference, as suggested by Edinger et al [54].

^s^SOL: sleep onset latency (min).

^t^10-minute difference in sleep onset latency, as suggested by Edinger et al [54].

^u^WASO: wake after sleep onset (min).

^v^15-minute difference in wake after sleep onset, as suggested by Edinger et al [54].

^w^When no minimal important differences are available, we chose 0.5 SD, as suggested by Norman et al [58].

^x^TST: total sleep time (min).

^y^15 minutes difference in total sleep time, as suggested by Edinger et al [54].

### Equivalence Meta-Analysis

As shown in Table 2, the 95% CI for total sleep disturbance was included in the prespecified equivalence interval for this outcome, and, based on the largest p-value of two one-sided tests, the null hypothesis of nonequivalence was rejected (*P*=.04). As shown in Table 2, when examining the various sleep outcomes, ipCBTI and eCBTI were statistically significantly equivalent for 25 (78%) out of 32 calculations. Furthermore, ipCBTI and eCBTI emerged as statistically significantly equivalent for all 3 secondary nonsleep outcomes at both time points (Table S4 in Multimedia Appendix 2).

### Publication Bias and Outliers

Inspecting the funnel plot and Egger's test for total sleep disturbance, which included data from all included studies (15/15, 100%), did not indicate publication bias (Egger test, *P*=.56; refer to the funnel plot in Figure S12 in Multimedia Appendix 2). Considering ESs, larger or smaller than 2 SDs beyond the pooled ES, revealed no outliers.

### Heterogeneity and Moderator Analyses

As seen in Table 2, heterogeneity analyses suggested that varying proportions of the variance in postintervention effects stem from between-study differences beyond random error. The *I*^2^ values were highest for the questionnaire-based sleep outcomes (62%-70%) and generally lower for the sleep diary and actigraphy-based outcomes (0%-31.2%). The data also suggested relatively high levels of heterogeneity for outcomes at follow-up. As shown in Table 3, when exploring possible explanations for the heterogeneity with meta-regression, 2 (18%) of the 11 analyzed moderators reached statistical significance. Differences between the proportions of dropouts in the eCBTI and ipCBTI moderated the between-group effects, with higher dropout rates in eCBTI compared with ipCBTI being associated with larger differences in favor of ipCBTI at both postintervention and follow-up, explaining 50% and 74% of the variation, respectively. Longer overall treatment duration was associated with larger differences in favor of ipCBTI compared with eCBTI at both time points. No statistically significant effects were found for the remaining moderators analyzed.

**Table 3:** Results of moderator analyses based on standardized mean differences (Hedges' *g*) in total self-reported sleep disturbance outcomes between in-person–delivered cognitive behavioral therapy for insomnia (ipCBTI) and eHealth cognitive behavioral therapy for insomnia (eCBTI) at postintervention and follow-up time points.

Moderator and time point		K^a^	Slope^b^ (95% CI)	*P* value^c^	*R* ^2^
**Mean sample age**					
	Postintervention	14	0.01 (–0.01 to 0.02)	.55	0.04
	Follow-up	12	–0.01 (–0.02 to 0.01)	.34	0.18
**Percentage of women**					
	Postintervention	15	0.00 (–0.01 to 0.01)	.71	0.03
	Follow-up	13	0.00 (–0.00 to 0.01)	.40	0.07
**Comorbid insomnia (reference: Insomnia as primary problem)**					
	Postintervention	15	0.16 (–0.31 to 0.62)	.51	0.04
	Follow-up	15	0.12 (–0.31 to 0.56)	.58	0.06
**Study dropout (%)**					
	Postintervention	13	0.00 (–0.02 to 0.03)	.78	0.00
	Follow-up	13	0.00 (–0.01 to 0.01)	.68	0.10
**eCBTI-ipCBTI dropout difference (%)^d^**					
	Postintervention	13	0.02 (0.00 to 0.03)	*.02* ^e^	0.50
	Follow-up	13	0.02 (0.01 to 0.03)	*.004*	0.74
**eCBTI therapist contact (reference: none)**					
	Postintervention	15	–0.07 (–0.51 to 0.36)	.74	0.01
	Follow-up	14	0.16 (–0.28 to 0.59)	.48	0.11
**Number of treatment sessions**					
	Postintervention	15	0.10 (–0.03 to 0.24)	.14	0.26
	Follow-up	14	0.11 (–0.03 to 0.25)	.13	0.32
**Treatment duration (weeks)**					
	Postintervention	15	0.18 (0.09 to 0.26)	*<.001*	0.93
	Follow-up	14	0.16 (0.08 to 0.24)	*<.001*	0.94
**Number of eCBTI components**					
	Postintervention	15	0.11 (–0.15 to 0.37)	.41	0.03
	Follow-up	14	0.18 (–0.06 to 0.42)	.14	0.15
**Number of ipCBTI components**					
	Postintervention	15	0.09 (–0.16 to 0.35)	.47	0.02
	Follow-up	14	0.17 (–0.07 to 0.41)	.18	0.11
**Time to follow-up (weeks)**					
	Follow-up	14	0.01 (–0.00 to 0.02)	.07	0.34

^a^K: number of studies in the analysis.

^b^Meta-regression (maximum likelihood method), conducted when K≥10.

^c^2-tailed *P* value.

^d^Difference in dropout (%) between conditions (eHealth cognitive behavioral therapy for insomnia [eCBTI] dropout minus in-person–delivered cognitive behavioral therapy for insomnia [ipCBTI] dropout). Positive values correspond to a higher dropout rate in eCBTI than in ipCBTI. Combined self-reported sleep quality outcomes include measures of insomnia severity (Insomnia Severity Index) and sleep quality (Pittsburgh Sleep Quality Index).

^e^Significant values (*P*<.05) are italicized.

### Results of Supplementary Bayesian Analyses

As presented in Table S5 in Multimedia Appendix 2, the Bayesian meta-analyses favored the alternative hypothesis of equivalence, that is, a zero difference between ipCBTI and eCBTI, for 4 (67%) out of 6 sleep outcomes at postintervention time points. The Bayes factors (BFs), that is, the probabilities of the alternative hypotheses relative to the null hypotheses, ranged from 1.7 (PSQI) to 9.9 (TST), indicating that a zero difference between ipCBTI and eCBTI is 1.7 to 9.9 times more likely than a nonzero difference. The level of evidence [93] ranged from anecdotal (BF=1-3) for PSQI to moderate (BF=3-10) for SOL, WASO, and TST. A 0 and nonzero difference for sleep efficiency appeared equally likely (BF=1.2). Insomnia severity assessed with the ISI was the only outcome for which the current evidence clearly favored a nonzero difference, with this result being 10.5 times more likely than the null hypothesis. Concerning heterogeneity, the data provided strong (BF=13.0) and anecdotal evidence (BF=2.3) for heterogeneous ISI and PSQI ESs, respectively. Nonheterogeneity was slightly more likely for the remaining outcomes (BF=1.9-3.5).

## Discussion

### Sleep Outcomes

When pooling the results of the 15 unique randomized trials directly comparing eCBTIs with ipCBTIs using conventional meta-analysis, the observed differences generally favored ipCBTI. Specifically, the postintervention results revealed statistically significant advantages for ipCBTI across several dimensions, including overall sleep disturbance (encompassing both insomnia severity and sleep quality), insomnia severity assessed independently, and sleep efficiency. While ipCBTI was statistically significantly superior to eCBTI for these outcomes, the magnitudes of these differences were modest, corresponding to small ESs (Hedges' *g*) and small mean, nonstandardized differences. For example, concerning the latter, the pooled mean differences in favor of ipCBTI for insomnia severity and sleep efficiency were only 1.8 points (on the ISI) and 1.9%, respectively. Furthermore, for total sleep disturbance and sleep efficiency, the CIs fell within the suggested equivalence margins of 0.5 SD and 5%, respectively [54].

Regarding the remaining self-reported sleep outcomes at postintervention, none yielded statistically significantly superior results in favor of either delivery type. Furthermore, with the exception of Hedges' *g* for the PSQI, all remaining analyses showed the 2 delivery types to be statistically significantly equivalent; that is, the CIs of the pooled effect parameter fell within the suggested equivalence margin for that parameter. The same general pattern was observed for the results obtained at the (on average) 21-week follow-up. On the basis of the available data, eCBTI and ipCBTI were statistically significantly equivalent for almost all self-reported sleep outcomes, except for the ES for sleep quality assessed with the PSQI and the mean difference in minutes for TST.

While equivalence indicates similar efficacy, if one only examines the between-group differences, it cannot be determined whether the equivalence stems from similar small or similar large improvements in both conditions. Therefore, we also calculated the within-group effects for each delivery format. The results revealed that both ipCBTI and eCBTI were associated with statistically significant within-condition improvements in all self-reported outcomes at both postintervention and follow-up. The largest effects were seen in both conditions for total sleep disturbance, insomnia severity, sleep quality, and sleep efficiency. Small-to-medium effects were observed for the remaining self-reported sleep outcomes. Therefore, it may be concluded that both delivery formats appear efficacious, displaying improvements at postintervention corresponding to 9- and 7-point reductions on the ISI, 12% and 10% improvements in sleep efficiency, 21- and 20-minute reductions in SOL, 24- and 20-minute reductions in WASO, and 21- to 16-minute increase in TST for ipCBTI and eCBTI, respectively. These effects are all clinically relevant and well beyond the suggested MIDs and minimal important changes for these outcomes, that is, a 6-point within-person change on the ISI [56] and 10, 15, and 15 minutes for SOL, WASO, and TST, respectively [54]. In addition, these clinically relevant, positive improvements were sustained over time, supporting previous findings that CBTI, regardless of the delivery format, yields robust long-term effects [28].

Only 3 (20%) of the 15 studies assessed sleep objectively, that is, with actigraphy. Despite the small number of studies, the results for sleep efficiency and SOL showed the 2 delivery formats to be statistically significantly equivalent. In contrast, the results for actigraphy-assessed TST appeared to be in favor of eCBTI, with eCBTI resulting in increased TST and ipCBTI in reduced TST. However, neither the conventional nor the equivalence analyses reached statistical significance. When examining the within-group effects, statistically significant improvements were seen for actigraphy-based SOL in the ipCBTI group. The remaining effects for TST and sleep efficiency failed to reach statistical significance. Such discrepancies between effects on self-reported and objectively assessed sleep outcomes, especially concerning estimates of sleep duration, are a well-recognized issue in sleep research and clinical practice [94].

### Nonsleep Outcomes

It is well known that insomnia can lead to various physical and mental symptoms, including increased levels of fatigue [95], and that it is a significant predictor of later onset of mental disorders such as depression and anxiety [8]. Therefore, we also explored the effects on the secondary nonsleep outcomes of fatigue, anxiety, and depression. The 2 delivery formats of CBTI were statistically significantly equivalent in their effects on these symptoms, and both yielded statistically significant medium-to-large within-condition improvements of almost identical magnitude in all 3 outcomes. Thus, our results add to the more general findings that CBTI may not only improve insomnia itself but also associated psychological and physical symptoms such as depression, anxiety, and fatigue [96-98], with our findings indicating that both delivery formats appear equally efficacious in reducing these symptoms.

### Heterogeneity and Its Sources

When exploring possible heterogeneity of the postintervention effects, the relatively large *I*^2^ statistics observed for both insomnia severity and sleep quality and the combined outcome of total sleep disturbance indicate that a considerable proportion of the variance is due to systematic differences between the study characteristics. In contrast, the differences in effects on sleep diary outcomes displayed little or no signs of heterogeneity. When we investigated possible sources of heterogeneity for the combined total sleep disturbance outcome, 2 study characteristics emerged as statistically significant moderators at both postintervention and follow-up.

First, higher dropout rates in eCBTI than in ipCBTI were significantly associated with larger differences in favor of ipCBTI in effect on total sleep disturbance. At postintervention time points, on average, 30% more participants in eCBTI had dropped out compared to ipCBTI. While we do not know the reasons for the higher dropout rates in eCBTI, this factor, which explained between 50% and 74% of the variance in between-condition differences in effect, could represent an important, potentially modifiable factor that needs to be addressed if the efficacy of eCBTI is to be further increased. While the research on adherence to electronically adapted interventions for insomnia is still limited, studies in this [99] and other clinical populations suggest that common factors influencing dropout and adherence across such interventions include engagement and motivation; technical issues and usability; and demographic factors such as age, educational level, and digital literacy [100,101]. Second, while there was no difference in the mean duration of the 2 delivery formats, interventions with longer duration favored ipCBTI. The moderating effect of intervention duration persisted when adjusting for study dropout. We have no clear explanation for this finding, but longer treatment duration may allow for increased trust and improved therapeutic alliance in personally delivered CBTI, which, in turn, will increase the effect.

None of the remaining moderators analyzed reached statistical significance, including demographic characteristics such as mean sample age and percentage of women in the sample; study characteristics such as time to follow-up; and treatment characteristics such as therapist contact versus no contact, the number of treatment sessions, and the number of CBTI components. Some of the nonsignificant results could be viewed as surprising. For example, one might have expected larger differences between ipCBTI and fully automated eCBTI than between ipCBTI and eCBTI with some degree of therapist contact. One would also have expected age to play a role, for example, that older sample age would be associated with larger between-condition differences. Possible reasons for nonsignificant findings could be insufficient between-study variation, for example, in sample age, and inadequate statistical power due to the relatively small number of studies. Further research is needed to identify the common and different factors associated with the increased efficacy of the 2 delivery formats.

### Limitations

Our findings should be interpreted cautiously for several reasons.

First, the interpretability is challenged by between-study heterogeneity, for example, by considerable between-study differences in eCBTI formats, with some eCBTIs delivered with direct therapist contact via telephone or videoconferencing; some delivered on the web with asynchronous therapist contact, for example, through email; and others offered as fully automated programs. While we attempted to explore the possible moderating role of such variations and found no indication of a moderating effect of the degree of therapist contact involved, the relatively small number of studies may have limited our ability to identify the influence of such characteristics.

Second, as demonstrated by the results of the Bayesian analyses, the small number of studies restricts the strength of the evidence. While the currently available evidence favored equivalent effects for 4 (67%) out of 6 outcomes (sleep quality, SOL, WASO, and TST), the level of evidence was weak (ie, anecdotal) to moderate. The evidence for sleep efficiency was inconclusive, and while the level of evidence for a nonzero difference in favor of ipCBTI was characterized as *strong*, the BF was only just above the lower limit (ie, ≥10) [93].

Third, interpreting the differences between eCBTI and ipCBTI as equivalent or nonequivalent clearly depends on the chosen equivalence margins. While we chose the MIDs suggested in the literature, for example, 2.6 points, 4.4 points, 5%, 10 minutes, 15 minutes, and 15 minutes for ISI, PSQI, sleep efficiency, SOL, WASO, and TST, respectively [48,54,55], specific MIDs have not been identified for all the corresponding ESs. While we used the 0.5 SD suggested in the literature [54,58], the clinical relevance of this value has yet to be established for several of the sleep outcomes investigated in this review.

Finally, assessed with the revised Cochrane Risk of Bias tool [51,102], one-third of the studies were characterized as having an overall *high risk* of bias, and the remaining two-thirds were characterized as having *some concerns*. Among the main reasons for these categorizations were high rates of missing outcome data and the use of self-reported outcomes. These issues cannot easily be amended. For example, it is not too surprising that behavioral interventions, in general, and eHealth interventions, in particular, have higher dropout rates than pharmacological trials. In addition, while sleep characteristics such as SOL, WASO, and TST can be assessed with both self-report and objective measures, insomnia is inherently a subjective outcome, which can be evaluated only with self-report. Furthermore, ensuring blinding is another factor that is difficult to obtain with behavioral interventions and not possible when comparing in-person and electronically delivered interventions. Other reasons for the identified risks of bias can be addressed more easily, including the failure to include sensitivity analyses correcting for missing outcome data and insufficient preregistering of analytical plans.

### Conclusions

This, to our knowledge, first systematic review and meta-analysis of randomized head-to-head comparisons of eCBTI and ipCBTI suggests that while the effects tended to be in favor of the latter, the mean differences were generally of small magnitudes, with several approaching 0. Furthermore, the 2 CBTI delivery formats were statistically significantly equivalent for most outcomes examined. Statistically significant equivalence means that the CIs of the differences fell within the prespecified equivalence margins, with the latter being based on the minimal clinically relevant differences suggested in the literature for each of the outcomes in question. Importantly, when examining the within-condition effects, both delivery formats yielded large and clinically relevant effects on most outcomes, including the nonsleep outcomes of fatigue, anxiety, and depression. Although the results should be interpreted cautiously due to the currently limited evidence base, they support eCBTI, including fully automated programs, as clinically relevant alternatives to ipCBTI. These results are promising for people with insomnia, given the challenges of meeting population needs with conventional treatment formats.

## Appendix

Multimedia Appendix 1 Completed PRISMA (Preferred Reporting Items for Systematic Reviews and Meta-Analysis) 2020 checklist.

Multimedia Appendix 2 Tables S1-S5 provide the detailed search strategy, list of excluded studies, within-group pre-post effects of in-person–delivered cognitive behavioral therapy for insomnia (ipCBTI) and eHealth cognitive behavioral therapy for insomnia (eCBTI), superiority and equivalence analyses of secondary non-sleep outcomes, supplementary Bayesian meta-analyses, and risk of bias assessments. Figures S1-S12 present forest plots of postintervention differences between effects of eCBTI and ipCBTI on primary and secondary sleep outcomes.

## Abbreviations

BF: Bayes factor
CBTI: cognitive behavioral therapy for insomnia
eCBTI: eHealth cognitive behavioral therapy for insomnia
ES: effect size
ipCBTI: in-person–delivered cognitive behavioral therapy for insomnia
ISI: Insomnia Severity Index
MID: minimal important difference
PICO: Population, Intervention, Comparison, and Outcome
PRISMA: Preferred Reporting Items for Systematic Reviews and Meta-Analysis
PSQI: Pittsburgh Sleep Quality Index
SOL: sleep onset latency
TST: total sleep time
WASO: wake after sleep onset

## References

[1] Morin CM, Jarrin DC (2022). Epidemiology of insomnia: prevalence, course, risk factors, and public health burden. Sleep Med Clin.

[2] Javaheri S, Redline S (2017). Insomnia and risk of cardiovascular disease. Chest.

[3] Sofi F, Cesari F, Casini A, Macchi C, Abbate R, Gensini GF (2014). Insomnia and risk of cardiovascular disease: a meta-analysis. Eur J Prev Cardiol.

[4] Cohen S, Doyle WJ, Alper CM, Janicki-Deverts D, Turner RB (2009). Sleep habits and susceptibility to the common cold. Arch Intern Med.

[5] Patel SR, Malhotra A, Gao X, Hu FB, Neuman MI, Fawzi WW (2012). A prospective study of sleep duration and pneumonia risk in women. Sleep.

[6] de Almondes KM, Costa MV, Malloy-Diniz LF, Diniz BS (2016). Insomnia and risk of dementia in older adults: systematic review and meta-analysis. J Psychiatr Res.

[7] Shi L, Chen SJ, Ma MY, Bao YP, Han Y, Wang YM, Shi J, Vitiello MV, Lu L (2018). Sleep disturbances increase the risk of dementia: a systematic review and meta-analysis. Sleep Med Rev.

[8] Hertenstein E, Feige B, Gmeiner T, Kienzler C, Spiegelhalder K, Johann A, Jansson-Fröjmark M, Palagini L, Rücker G, Riemann D, Baglioni C (2019). Insomnia as a predictor of mental disorders: a systematic review and meta-analysis. Sleep Med Rev.

[9] Hom MA, Hames JL, Bodell LP, Buchman-Schmitt JM, Chu C, Rogers ML, Chiurliza B, Michaels MS, Ribeiro JD, Nadorff MR, Winer ES, Lim IC, Rudd MD, Joiner TE (2017). Investigating insomnia as a cross-sectional and longitudinal predictor of loneliness: findings from six samples. Psychiatry Res.

[10] Ben Simon E, Walker MP (2018). Sleep loss causes social withdrawal and loneliness. Nat Commun.

[11] Cappuccio FP, D'Elia L, Strazzullo P, Miller MA (2010). Sleep duration and all-cause mortality: a systematic review and meta-analysis of prospective studies. Sleep.

[12] Liu TZ, Xu C, Rota M, Cai H, Zhang C, Shi MJ, Yuan RX, Weng H, Meng XY, Kwong JS, Sun X (2017). Sleep duration and risk of all-cause mortality: a flexible, non-linear, meta-regression of 40 prospective cohort studies. Sleep Med Rev.

[13] Daley M, Morin CM, LeBlanc M, Grégoire JP, Savard J (2009). The economic burden of insomnia: direct and indirect costs for individuals with insomnia syndrome, insomnia symptoms, and good sleepers. Sleep.

[14] Daley M, Morin CM, LeBlanc M, Grégoire JP, Savard J, Baillargeon L (2009). Insomnia and its relationship to health-care utilization, work absenteeism, productivity and accidents. Sleep Med.

[15] Curcio G, Ferrara M, de Gennaro L (2006). Sleep loss, learning capacity and academic performance. Sleep Med Rev.

[16] Trauer JM, Qian MY, Doyle JS, Rajaratnam SM, Cunnington D (2015). Cognitive behavioral therapy for chronic insomnia: a systematic review and meta-analysis. Ann Intern Med.

[17] Morin CM, Benca R (2012). Chronic insomnia. The Lancet.

[18] Kripke DF (2016). Hypnotic drug risks of mortality, infection, depression, and cancer: but lack of benefit. F1000Res.

[19] Edinger JD, Arnedt JT, Bertisch SM, Carney CE, Harrington JJ, Lichstein KL, Sateia MJ, Troxel WM, Zhou ES, Kazmi U, Heald JL, Martin JL (2021). Behavioral and psychological treatments for chronic insomnia disorder in adults: an American Academy of Sleep Medicine clinical practice guideline. J Clin Sleep Med.

[20] Riemann D, Baglioni C, Bassetti C, Bjorvatn B, Dolenc Groselj L, Ellis JG, Espie CA, Garcia-Borreguero D, Gjerstad M, Gonçalves M, Hertenstein E, Jansson-Fröjmark M, Jennum PJ, Leger D, Nissen C, Parrino L, Paunio T, Pevernagie D, Verbraecken J, Weeß HG, Wichniak A, Zavalko I, Arnardottir ES, Deleanu O, Strazisar B, Zoetmulder M, Spiegelhalder K (2017). European guideline for the diagnosis and treatment of insomnia. J Sleep Res.

[21] Morin CM, Bei B, Bjorvatn B, Poyares D, Spiegelhalder K, Wing YK (2023). World sleep society international sleep medicine guidelines position statement endorsement of "behavioral and psychological treatments for chronic insomnia disorder in adults: an American Academy of sleep medicine clinical practice guidelines". Sleep Med.

[22] Spielman AJ, Saskin P, Thorpy MJ (1987). Treatment of chronic insomnia by restriction of time in bed. Sleep.

[23] Bootzin R, Perlis M, Perlis M, Aloia M, Kuhn B (2011). Stimulus-control therapy. Behavioral Treatments for Sleep Disorders: A Volume in Practical Resources for the Mental Health Professional.

[24] Freedman R, Papsdorf JD (1976). Biofeedback and progressive relaxation treatment of sleep-onset insomnia. Biofeedback Self Regul.

[25] Belanger L, Savard J, Morin CM (2006). Clinical management of insomnia using cognitive therapy. Behav Sleep Med.

[26] Bootzin RR, Perlis ML (1992). Nonpharmacologic treatments of insomnia. J Clin Psychiatry.

[27] van Straten A, van der Zweerde T, Kleiboer A, Cuijpers P, Morin CM, Lancee J (2018). Cognitive and behavioral therapies in the treatment of insomnia: a meta-analysis. Sleep Med Rev.

[28] van der Zweerde T, Bisdounis L, Kyle SD, Lancee J, van Straten A (2019). Cognitive behavioral therapy for insomnia: a meta-analysis of long-term effects in controlled studies. Sleep Med Rev.

[29] Selvanathan J, Pham C, Nagappa M, Peng PW, Englesakis M, Espie CA, Morin CM, Chung F (2021). Cognitive behavioral therapy for insomnia in patients with chronic pain - a systematic review and meta-analysis of randomized controlled trials. Sleep Med Rev.

[30] Squires LR, Rash JA, Fawcett J, Garland SN (2022). Systematic review and meta-analysis of cognitive-behavioural therapy for insomnia on subjective and actigraphy-measured sleep and comorbid symptoms in cancer survivors. Sleep Med Rev.

[31] Zhang Y, Ren R, Yang L, Zhang H, Shi Y, Shi J, Sanford LD, Lu L, Vitiello MV, Tang X (2022). Comparative efficacy and acceptability of psychotherapies, pharmacotherapies, and their combination for the treatment of adult insomnia: a systematic review and network meta-analysis. Sleep Med Rev.

[32] Koffel E, Bramoweth AD, Ulmer CS (2018). Increasing access to and utilization of cognitive behavioral therapy for insomnia (CBT-I): a narrative review. J Gen Intern Med.

[33] Conroy DA, Ebben MR (2015). Referral practices for cognitive behavioral therapy for insomnia: a survey study. Behav Neurol.

[34] Roach M, Juday T, Tuly R, Chou JW, Jena AB, Doghramji PP (2021). Challenges and opportunities in insomnia disorder. Int J Neurosci.

[35] Cheng SK, Dizon J (2012). Computerised cognitive behavioural therapy for insomnia: a systematic review and meta-analysis. Psychother Psychosom.

[36] Zachariae R, Lyby MS, Ritterband LM, O'Toole MS (2016). Efficacy of internet-delivered cognitive-behavioral therapy for insomnia - a systematic review and meta-analysis of randomized controlled trials. Sleep Med Rev.

[37] Ritterband LM, Thorndike FP, Gonder-Frederick LA, Magee JC, Bailey ET, Saylor DK, Morin CM (2009). Efficacy of an internet-based behavioral intervention for adults with insomnia. Arch Gen Psychiatry.

[38] Kaldo V, Jernelöv S, Blom K, Ljótsson B, Brodin M, Jörgensen M, Kraepelien M, Rück C, Lindefors N (2015). Guided internet cognitive behavioral therapy for insomnia compared to a control treatment - a randomized trial. Behav Res Ther.

[39] Zachariae R, Amidi A, Damholdt MF, Clausen CD, Dahlgaard J, Lord H, Thorndike FP, Ritterband LM (2018). Internet-delivered cognitive-behavioral therapy for insomnia in breast cancer survivors: a randomized controlled trial. J Natl Cancer Inst.

[40] Hasan F, Tu YK, Yang CM, James Gordon C, Wu D, Lee HC, Yuliana LT, Herawati L, Chen TJ, Chiu HY (2022). Comparative efficacy of digital cognitive behavioral therapy for insomnia: a systematic review and network meta-analysis. Sleep Med Rev.

[41] Simon L, Steinmetz L, Feige B, Benz F, Spiegelhalder K, Baumeister H (2023). Comparative efficacy of onsite, digital, and other settings for cognitive behavioral therapy for insomnia: a systematic review and network meta-analysis. Sci Rep.

[42] Forma F, Pratiwadi R, El-Moustaid F, Smith N, Thorndike F, Velez F (2022). Network meta-analysis comparing the effectiveness of a prescription digital therapeutic for chronic insomnia to medications and face-to-face cognitive behavioral therapy in adults. Curr Med Res Opin.

[43] Lakens D, Scheel AM, Isager PM (2018). Equivalence testing for psychological research: a tutorial. Adv Method Pract Psychol Sci.

[44] Rogers JL, Howard KI, Vessey JT (1993). Using significance tests to evaluate equivalence between two experimental groups. Psychol Bull.

[45] Jaeschke R, Singer J, Guyatt GH (1989). Measurement of health status. Ascertaining the minimal clinically important difference. Control Clin Trials.

[46] Page MJ, McKenzie JE, Bossuyt PM, Boutron I, Hoffmann TC, Mulrow CD, Shamseer L, Tetzlaff JM, Akl EA, Brennan SE, Chou R, Glanville J, Grimshaw JM, Hróbjartsson A, Lalu MM, Li T, Loder EW, Mayo-Wilson E, McDonald S, McGuinness LA, Stewart LA, Thomas J, Tricco AC, Welch VA, Whiting P, Moher D (2021). The PRISMA 2020 statement: an updated guideline for reporting systematic reviews. BMJ.

[47] Straus SE (2005). Evidence-Based Medicine: How to Practice and Teach EBM.

[48] Bastien CH, Vallières A, Morin CM (2001). Validation of the Insomnia Severity Index as an outcome measure for insomnia research. Sleep Med.

[49] Buysse DJ, Reynolds CF 3rd, Monk TH, Berman SR, Kupfer DJ (1989). The Pittsburgh Sleep Quality Index: a new instrument for psychiatric practice and research. Psychiatry Res.

[50] Covidence systematic review software. Veritas Health Innovation.

[51] Sterne JA, Savović J, Page MJ, Elbers RG, Blencowe NS, Boutron I, Cates CJ, Cheng HY, Corbett MS, Eldridge SM, Emberson JR, Hernán MA, Hopewell S, Hróbjartsson A, Junqueira DR, Jüni P, Kirkham JJ, Lasserson T, Li T, McAleenan A, Reeves BC, Shepperd S, Shrier I, Stewart LA, Tilling K, White IR, Whiting PF, Higgins JP (2019). RoB 2: a revised tool for assessing risk of bias in randomised trials. BMJ.

[52] Soh HL, Ho RC, Ho CS, Tam WW (2020). Efficacy of digital cognitive behavioural therapy for insomnia: a meta-analysis of randomised controlled trials. Sleep Med.

[53] Hedges LV, Olkin I (1985). Statistical Methods for Meta-Analysis.

[54] Edinger JD, Arnedt JT, Bertisch SM, Carney CE, Harrington JJ, Lichstein KL, Sateia MJ, Troxel WM, Zhou ES, Kazmi U, Heald JL, Martin JL (2021). Behavioral and psychological treatments for chronic insomnia disorder in adults: an American Academy of Sleep Medicine systematic review, meta-analysis, and GRADE assessment. J Clin Sleep Med.

[55] Longo UG, Berton A, de Salvatore S, Piergentili I, Casciani E, Faldetta A, de Marinis MG, Denaro V (2021). Minimal clinically important difference and patient acceptable symptom state for the Pittsburgh Sleep Quality Index in patients who underwent rotator cuff tear repair. Int J Environ Res Public Health.

[56] Yang M, Morin CM, Schaefer K, Wallenstein GV (2009). Interpreting score differences in the Insomnia Severity Index: using health-related outcomes to define the minimally important difference. Curr Med Res Opin.

[57] Cuijpers P, Turner EH, Koole SL, van Dijke A, Smit F (2014). What is the threshold for a clinically relevant effect? the case of major depressive disorders. Depress Anxiety.

[58] Norman GR, Sloan JA, Wyrwich KW (2003). Interpretation of changes in health-related quality of life: the remarkable universality of half a standard deviation. Med Care.

[59] Cooper H, Hedges LV, Valentine JC (2009). The Handbook of Research Synthesis and Meta-Analysis.

[60] Higgins JP, Thompson SG, Deeks JJ, Altman DG (2003). Measuring inconsistency in meta-analyses. BMJ.

[61] IntHout J, Ioannidis JP, Rovers MM, Goeman JJ (2016). Plea for routinely presenting prediction intervals in meta-analysis. BMJ Open.

[62] Egger M, Davey Smith G, Schneider M, Minder C (1997). Bias in meta-analysis detected by a simple, graphical test. BMJ.

[63] Duval S, Tweedie R (2000). Trim and fill: a simple funnel-plot-based method of testing and adjusting for publication bias in meta-analysis. Biometrics.

[64] Borenstein M, Hedges LV, Higgins JP, Rothstein H (2021). Comprehensive meta‐analysis software. Introduction to Meta‐Analysis.

[65] Gronau QF, van Erp S, Heck DW, Cesario J, Jonas KJ, Wagenmakers EJ (2017). A Bayesian model-averaged meta-analysis of the power pose effect with informed and default priors: the case of felt power. Compr Results Soc Psychol.

[66] Wasserstein RL, Lazar NA (2016). The ASA statement on p-values: context, process, and purpose. Am Stat.

[67] van Erp S, Verhagen J, Grasman R, Wagenmakers EJ (2017). Estimates of between-study heterogeneity for 705 meta-analyses reported in psychological bulletin from 1990-2013. J Open Psychol Data.

[68] JASP 0.17.1 (Windows 64 Bit). JASP Team.

[69] Currie SR, Clark S, Hodgins DC, El-Guebaly N (2004). Randomized controlled trial of brief cognitive-behavioural interventions for insomnia in recovering alcoholics. Addiction.

[70] Bastien CH, Morin CM, Ouellet MC, Blais FC, Bouchard S (2004). Cognitive-behavioral therapy for insomnia: comparison of individual therapy, group therapy, and telephone consultations. J Consult Clin Psychol.

[71] Savard J, Ivers H, Savard MH, Morin CM (2014). Is a video-based cognitive behavioral therapy for insomnia as efficacious as a professionally administered treatment in breast cancer? results of a randomized controlled trial. Sleep.

[72] Savard J, Ivers H, Savard M, Morin CM (2016). Long-term effects of two formats of cognitive behavioral therapy for insomnia comorbid with breast cancer. Sleep.

[73] Blom K, Tarkian Tillgren H, Wiklund T, Danlycke E, Forssén M, Söderström A, Johansson R, Hesser H, Jernelöv S, Lindefors N, Andersson G, Kaldo V (2015). Internet-vs. group-delivered cognitive behavior therapy for insomnia: a randomized controlled non-inferiority trial. Behav Res Ther.

[74] de Bruin EJ, Bögels SM, Oort FJ, Meijer AM (2015). Efficacy of cognitive behavioral therapy for insomnia in adolescents: a randomized controlled trial with internet therapy, group therapy and a waiting list condition. Sleep.

[75] de Bruin EJ, Bögels SM, Oort FJ, Meijer AM (2018). Improvements of adolescent psychopathology after insomnia treatment: results from a randomized controlled trial over 1 year. J Child Psychol Psychiatry.

[76] Lancee J, van Straten A, Morina N, Kaldo V, Kamphuis JH (2016). Guided online or face-to-face cognitive behavioral treatment for insomnia: a randomized wait-list controlled trial. Sleep.

[77] Taylor DJ, Peterson AL, Pruiksma KE, Young-McCaughan S, Nicholson K, Mintz J (2017). Internet and in-person cognitive behavioral therapy for insomnia in military personnel: a randomized clinical trial. Sleep.

[78] Laurel Franklin C, Walton JL, Raines AM, Chambliss JL, Corrigan SA, Cuccurullo LA, Petersen NJ, Thompson KE (2017). Pilot study comparing telephone to in-person delivery of cognitive-behavioural therapy for trauma-related insomnia for rural veterans. J Telemed Telecare.

[79] Gieselmann A, Pietrowsky R (2019). The effects of brief chat-based and face-to-face psychotherapy for insomnia: a randomized waiting list controlled trial. Sleep Med.

[80] Gehrman P, Barilla H, Medvedeva E, Bellamy S, O'Brien E, Kuna ST (2020). Randomized trial of telehealth delivery of cognitive-behavioral treatment for insomnia vs. in-person treatment in veterans with PTSD. J Affect Disord Rep.

[81] Arnedt JT, Conroy DA, Mooney A, Furgal A, Sen A, Eisenberg D (2021). Telemedicine versus face-to-face delivery of cognitive behavioral therapy for insomnia: a randomized controlled noninferiority trial. Sleep.

[82] Gehrman P, Gunter P, Findley J, Frasso R, Weljie AM, Kuna ST, Kayser MS (2021). Randomized noninferiority trial of telehealth delivery of cognitive behavioral treatment of insomnia compared to in-person care. J Clin Psychiatry.

[83] Kallestad H, Scott J, Vedaa Ø, Lydersen S, Vethe D, Morken G, Stiles TC, Sivertsen B, Langsrud K (2021). Mode of delivery of cognitive behavioral therapy for insomnia: a randomized controlled non-inferiority trial of digital and face-to-face therapy. Sleep.

[84] Wong KY, Chung KF, Au CH (2021). Low-intensity cognitive behavioral therapy for insomnia as the entry of the stepped-care model in the community: a randomized controlled trial. Behav Sleep Med.

[85] Chan NY, Lam SP, Zhang J, Chan JW, Yu MM, Suh S, Yang CM, Okajima I, Li AM, Wing YK, Li SX (2022). Efficacy of email-delivered versus face-to-face group cognitive behavioral therapy for insomnia in youths: a randomized controlled trial. J Adolesc Health.

[86] Zigmond AS, Snaith RP (1983). The hospital anxiety and depression scale. Acta Psychiatr Scand.

[87] Beck AT, Steer RA, Brown GK (1995). BDI-II, Beck Depression Inventory: Manual.

[88] Kroenke K, Spitzer RL, Williams JB (2001). The PHQ-9: validity of a brief depression severity measure. J Gen Intern Med.

[89] Radloff LS (2016). The CES-D scale: a self-report depression scale for research in the general population. Appl Psychol Meas.

[90] Spitzer RL, Kroenke K, Williams JB, Löwe B (2006). A brief measure for assessing generalized anxiety disorder: the GAD-7. Arch Intern Med.

[91] Smets EM, Garssen B, Bonke B, de Haes JC (1995). The Multidimensional Fatigue Inventory (MFI) psychometric qualities of an instrument to assess fatigue. J Psychosom Res.

[92] Carney CE, Buysse DJ, Ancoli-Israel S, Edinger JD, Krystal AD, Lichstein KL, Morin CM (2012). The consensus sleep diary: standardizing prospective sleep self-monitoring. Sleep.

[93] Jeffreys H (1984). Theory of Probability, Third Edition.

[94] Buysse DJ (2014). Sleep health: can we define it? does it matter?. Sleep.

[95] Huang L, Zhu W, Li N, Zhang B, Dai W, Li S, Xu H (2024). Functions and mechanisms of adenosine and its receptors in sleep regulation. Sleep Med.

[96] Ballesio A, Aquino MR, Feige B, Johann AF, Kyle SD, Spiegelhalder K, Lombardo C, Rücker G, Riemann D, Baglioni C (2018). The effectiveness of behavioural and cognitive behavioural therapies for insomnia on depressive and fatigue symptoms: a systematic review and network meta-analysis. Sleep Med Rev.

[97] Ye YY, Zhang YF, Chen J, Liu J, Li XJ, Liu YZ, Lang Y, Lin L, Yang XJ, Jiang XJ (2015). Internet-based cognitive behavioral therapy for insomnia (ICBT-i) improves comorbid anxiety and depression-a meta-analysis of randomized controlled trials. PLoS One.

[98] Tang NK, Lereya ST, Boulton H, Miller MA, Wolke D, Cappuccio FP (2015). Nonpharmacological treatments of insomnia for long-term painful conditions: a systematic review and meta-analysis of patient-reported outcomes in randomized controlled trials. Sleep.

[99] Sanchez-Ortuno MM, Pecune F, Coelho J, Micoulaud-Franchi JA, Salles N, Auriacombe M, Serre F, Levavasseur Y, de Sevin E, Sagaspe P, Philip P (2023). Predictors of users' adherence to a fully automated digital intervention to manage insomnia complaints. J Am Med Inform Assoc.

[100] Pedersen DH, Mansourvar M, Sortsø C, Schmidt T (2019). Predicting dropouts from an electronic health platform for lifestyle interventions: analysis of methods and predictors. J Med Internet Res.

[101] Alfonsson S, Olsson E, Hursti T (2016). Motivation and treatment credibility predicts dropout, treatment adherence, and clinical outcomes in an internet-based cognitive behavioral relaxation program: a randomized controlled trial. J Med Internet Res.

[102] McGuinness LA, Higgins JP (2021). Risk-of-bias VISualization (robvis): an R package and Shiny web app for visualizing risk-of-bias assessments. Res Synth Methods.

