# Supplementary material for: Efficacy of eHealth Versus In-Person Cognitive Behavioral Therapy for Insomnia: Systematic Review and Meta-Analysis of Equivalence
Source: JMIR Ment Health. 2024 Aug 26;11:e58217. doi: 10.2196/58217 (PMC11384180; doi:10.2196/58217)
Supplement: Multimedia Appendix 2 [file mental_v11i1e58217_app2.pdf]

Multimedia appendix 2. Efficacy of eHealth vs. in-person cognitive-behavioral therapy for insomnia: A systematic review and meta-analysis of equivalence

Contents

Table S1: Search strings 2
Table S2: List of studies excluded after full text screening with reasons for exclusion 4
Table S3: Within-group pre-post effects of ipCBTI and eCBTI on primary sleep outcomes and secondary sleep and non-sleep outcomes 8
Table S4: Secondary non-sleep outcomes: Fatigue, anxiety, and depression 11
Table S5: Results of supplementary Bayesian meta-analyses of the post-intervention results 12
Figure S1: Risk of Bias (RoB 2) 13
Figure S2: Forest plot of post-intervention mean differences (points) between effects of eCBTI and ipCBTI on insomnia severity (ISI) 14
Figure S3: Forest plot of post-intervention differences (Hedges's g) between effects of eCBTI and ipCBTI on insomnia severity (ISI) 15
Figure S4: Forest plot of post-intervention mean differences (points) between effects of eCBTI and ipCBTI on sleep quality (PSQI) 16
Figure S5: Forest plot of post-intervention differences (Hedges's g) between effects of eCBTI and ipCBTI on sleep quality (PSQI) 17
Figure S6: Forest plot of post-intervention differences (Hedges's g) between effects of eCBTI and ipCBTI on diary-based sleep efficiency 18
Figure S7: Forest plot of post-intervention mean differences (minutes) between effects of eCBTI and ipCBTI on diary-based sleep onset latency (SOL) 19
Figure S8: Forest plot of post-intervention differences (Hedges's g) between effects of eCBTI and ipCBTI on diary-based sleep onset latency (SOL) 20
Figure S9: Forest plot of post-intervention mean differences (minutes) between effects of eCBTI and ipCBTI on diary-based wake after sleep onset (WASO) 21
Figure S10: Forest plot of post-intervention differences (Hedges's g) between effects of eCBTI and ipCBTI on diary-based wake after sleep onset (WASO) 22
Figure S11: Forest plot of post-intervention differences (Hedges's g) between effects of eCBTI and ipCBTI on diary-based total sleep time (TST) 23
Figure S12: Funnel plot of post-intervention differences (Hedges's g) between effects of eCBTI and ipCBTI on total sleep disturbance 24
References to figures and tables 25

Table S1: Search strings

CINAHL: ((MH "Dyssomnias+") OR (MH "Sleep Disorders+") OR (MH "Insomnia+") OR insomnia OR "sleep disturbance" OR "sleep disturbances" OR "sleep disorder" OR "sleep disorders" OR "disordered sleep" OR "disturbed sleep" OR "Sleep Initiation and Maintenance Disorders" OR "Sleep Wake Disorders" OR dyssomnia OR Dyssomnias) AND ((MH "Cognitive Therapy+") OR "cognitive behavior" OR "cognitive behaviour" OR "cognitive behavioral" OR "cognitive behavioural" OR CBT OR "CBT I") AND ((MH "Internet+") OR (MH "Telemedicine+") OR (MH "Telehealth+") OR eHealth OR "e health" OR telehealth OR "tele health" OR telemedicine OR "tele medicine" OR internet* OR "web based" OR webbased OR online OR digital* OR computer* OR app OR application* OR phone* OR smartphone* OR "smart phone" OR "smart phones" OR mobile OR "eCBT I" OR "e CBT I" OR "Internet-Based Intervention" OR Telephone OR "Cell Phone" OR video* OR audi*)

PsycInfo: (MAINSUBJECT.EXACT.EXPLODE("Insomnia") OR MAINSUBJECT.EXACT.EXPLODE("Sleep Wake Disorders") OR (insomnia OR "sleep disturbance" OR "sleep disturbances" OR "sleep disorder" OR "sleep disorders" OR "disordered sleep" OR "disturbed sleep" OR "Sleep Initiation and Maintenance Disorders" OR "Sleep Wake Disorders" OR dyssomnia OR Dyssomnias)) AND (MAINSUBJECT.EXACT.EXPLODE("Cognitive Behavior Therapy") OR ("cognitive behavior" OR "cognitive behaviour" OR "cognitive behavioral" OR "cognitive behavioural" OR CBT OR "CBT I")) AND ((MAINSUBJECT.EXACT.EXPLODE("Telemedicine") OR MAINSUBJECT.EXACT.EXPLODE("Internet") OR MAINSUBJECT.EXACT.EXPLODE("Digital Interventions") OR (MAINSUBJECT.EXACT.EXPLODE("Smartphone Use") OR MAINSUBJECT.EXACT("Smartphones") OR MAINSUBJECT.EXACT.EXPLODE("Mobile Phones"))) OR (eHealth OR "e health" OR telehealth OR "tele health" OR telemedicine OR "tele medicine" OR internet* OR "web based" OR webbased OR online OR digital* OR computer* OR app OR application* OR phone* OR smartphone* OR "smart phone" OR "smart phones" OR mobile OR "eCBT I" OR "e CBT I" OR "Internet-Based Intervention" OR Telephone OR "Cell Phone" OR video* OR audi*))

PubMed: ((((((insomnia OR "sleep disturbance" OR "sleep disturbances" OR "sleep disorder" OR "sleep disorders" OR "disturbed sleep" OR "disordered sleep") OR (Sleep Initiation and Maintenance Disorders[Mesh])) OR (Sleep Wake Disorders[Mesh])) OR (Dyssomnias[Mesh]))) AND (("cognitive behavior" OR "cognitive behaviour" OR "cognitive behavioral" OR "cognitive behavioural" OR CBT OR "CBT I") OR (Cognitive Behavioral Therapy[Mesh]))) AND (((((((eHealth OR "e health" OR telehealth OR "tele health" OR telemedicine OR "tele medicine" OR internet* OR "web based" OR webbased OR online OR digital* OR computer* OR app OR application* OR phone* OR smartphone* OR "smart phone" OR "smart phones" OR mobile OR "eCBT I" OR "e CBT I" OR video* OR audi*) OR (Telemedicine[Mesh])) OR (Internet[Mesh] OR (Internet-Based Intervention[Mesh])) OR

(Computers[Mesh])) OR (Mobile Applications[Mesh])) OR (Telephone[Mesh] OR Cell Phone[Mesh])) OR (Cell Phone Use[Mesh])) OR (Smartphone[Mesh]))

Embase: ('sleep disturbance'/exp OR 'sleep disturbance*' OR 'sleep disorder'/exp OR 'sleep disorder*' OR 'insomnia'/exp OR insomnia* OR 'disturbed sleep' OR 'disordered sleep') AND ('cognitive behavior' OR 'cognitive behaviour' OR 'cognitive behavioral' OR 'cognitive behavioural' OR cbt OR 'cbt i' OR 'cognitive behavioral therapy'/exp OR 'cognitive behavioral therapy') AND (ehealth OR 'e health' OR telehealth OR 'tele health' OR telemedicine OR 'tele medicine' OR internet* OR 'web based' OR webbased OR online OR digital* OR computer* OR app OR application* OR phone* OR smartphone* OR 'smart phone' OR 'smart phones' OR mobile OR 'ecbt i' OR 'e cbt i' OR video* OR audi* OR 'mobile application'/exp OR 'mobile application' OR 'mobile phone'/exp OR 'mobile phone' OR 'smartphone'/exp OR smartphone OR 'computer'/exp OR computer OR 'internet'/exp OR internet OR 'web-based intervention'/exp OR 'web-based intervention' OR 'telemedicine'/exp OR telemedicine OR 'telehealth'/exp OR telehealth)

Cochrane Library: https://www.cochranelibrary.com/advanced-search/search-manager?search=7350979

Table S2: List of studies excluded after full text screening with reasons for exclusion

	Reference	Reason for exclusion
1	Abdelaziz EM, Elsharkawy NB, Mohamed SM. Efficacy of Internet-based cognitive behavioral therapy on sleeping difficulties in menopausal women: A randomized controlled trial. Perspectives in Psychiatric Care. 2022 Oct;58(4):1907-17. PMID: 34931313. doi: 10.1111/ppc.13005.	No ipCBTI condition
2	Arizmendi BJ, Gress-Smith JL, Krieg C, Waddell J. Adapting Group CBT-I for Telehealth-to-Home With Military Veterans in Primary Care. Journal of Primary Care & Community Health. 2023 Jan-Dec;14:21501319221143722. PMID: 36625248. doi: 10.1177/21501319221143722.	Non-randomized allocation
3	Azimi M, Moradi A, Hasani J. Effectiveness of cognitive behavioral therapy for insomnia (traditional and Internet-based) on everyday memory of people with insomnia and comorbid depression. Advances in cognitive science. 2019 Jan;20(4):20-34. PMID: CN-01994578.	Other language than English
4	Azimi M, Moradi A, Hasani J. Effectiveness of cognitive behavioral therapy for insomnia and Internet-based cognitive behavioral therapy for insomnia on people with insomnia comorbid depression. Journal of Psychology. 2019;22(4):430-47.	Other language than English
5	Behrendt D, Ebert DD, Spiegelhalder K, Lehr D. Efficacy of a Self-Help Web-Based Recovery Training in Improving Sleep in Workers: Randomized Controlled Trial in the General Working Population. Journal of Medical Internet Research. 2020 Jan 7;22(1):e13346. PMID: 31909725. doi: 10.2196/13346.	No ipCBTI condition
6	Bei B, Asarnow LD, Krystal A, Edinger JD, Buysse DJ, Manber R. Treating insomnia in depression: Insomnia related factors predict long-term depression trajectories. Journal of Consulting and Clinical Psychology. 2018 Mar ;86(3):282-93. PMID: 2010400085; 2018-08989-006. doi: 10.1037/ccp0000282.	No eCBTI condition
7	Blom K, Jernelöv S, Rück C, Lindefors N, Kaldo V. Three-Year Follow-Up of Insomnia and Hypnotics after Controlled Internet Treatment for Insomnia. Sleep. 2016;39(6):1267-74. PMID: CN-01379899. doi: 10.5665/sleep.5850.	No ipCBTI condition
8	Chevalier LL, Fine E, Sharma A, Zhou ES, Recklitis CJ. Evaluating the Sleep Treatment Education Program (STEP-1): A single-session educational workshop addressing insomnia in cancer survivors. J Psychosoc Oncol. 2023;41(1):123-32. PMID: 35468047. doi: 10.1080/07347332.2022.2054750.	Non-randomized allocation
9	de bruin EJ, Meijer AM, Oort FJ, Bgels SM. Efficacy of internet and group administered cognitive behavioral therapy for insomnia in adolescents; A pilot study. Sleep and Biological Rhythms. 2011;9(4):259. doi: 10.1111/j.1479-8425.2011.00518.x.	Conference abstract or similar

10	de Bruin EJ, Oort FJ, Bögels SM, Meijer AM. Efficacy of internet and group-administered cognitive behavioral therapy for insomnia in adolescents: A pilot study. Behavioral Sleep Medicine. 2014 May;12(3):235-54. PMID: 1606032008; 2014-11731-006. doi: 10.1080/15402002.2013.784703.	Non-randomized allocation
11	de Bruin EJ, Oort FJ, Bogels SM, Meijer AM. Efficacy of cognitive behavior therapy in adolescents with insomnia: an RCT with group-CBTi, internet-CBTi and a waiting list condition. Journal of sleep research. 2014;23:42. PMID: CN-01066280. doi: 10.1111/jsr.12213.	Conference abstract or similar
12	de Bruin EJ, van Steensel FJ, Meijer AM. Cost-Effectiveness of Group and Internet Cognitive Behavioral Therapy for Insomnia in Adolescents: results from a Randomized Controlled Trial. Sleep. 2016;39(8):1571-81. PMID: CN-01411332. doi: 10.5665/sleep.6024.	Secondary analysis (and no outcomes of interest)
13	de Bruin E, Meijer AM, Oort FJ, Bögels SM. Effects of online and group administered cognitive behavioural therapy for insomnia on sleep, chronic sleep reduction and behavioural problems in adolescents: A pilot study. Journal of Sleep Research. 2012;21:352. doi: 10.1111/j.1365-2869.2012.01044.x.	Conference abstract or similar
14	de Bruin E, Van Steensel B, Meijer AM. Cost-effectiveness of group-and Internet cognitive behavioural therapy for insomnia in adolescents: results from a randomized controlled trial. Journal of sleep research. 2016;25:124. PMID: CN-01213328. doi: 10.1111/jsr.12446.	Conference abstract or similar
15	Derose SF, Rozema E, Chen A, Shen E, Hwang D, Manthena P. A population health approach to insomnia using internet-based cognitive behavioral therapy for insomnia. Journal of clinical sleep medicine. 2021;17(8):1675-84. PMID: CN-02274163. doi: 10.5664/jcsm.9280.	No outcomes of interest
16	Fales J, Law E, Claar R, Palermo T. Sleep outcomes in adolescents with chronic pain: Findings from a multi-site randomized clinical trial of webbased cognitive behavioral therapy for pediatric chronic pain. Journal of Pain. 2013;14(4):S99. doi: 10.1016/j.jpain.2013.01.735.	Conference abstract or similar
17	Felder JN, Epel ES, Neuhaus J, Krystal AD, Prather AA. Efficacy of Digital Cognitive Behavioral Therapy for the Treatment of Insomnia Symptoms Among Pregnant Women: A Randomized Clinical Trial. JAMA Psychiatry. 2020 May 1;77(5):484-92. PMID: 31968068. doi: 10.1001/jamapsychiatry.2019.4491.	No ipCBTI condition
18	Granberg RE, Heyer A, Gehrman PR, Gunter PW, Hoff NA, Guth A, et al. Patient and provider experiences with CBT-I administered in-person or via telemedicine: A randomized non-inferiority trial. Cogent Psychology. 2022 Jan;9(1):12. PMID: 2700773829; 2022-35987-001. doi: https://doi.org/10.1080/23311908.2022.2038936.	No outcomes of interest
19	Holmqvist M, Vincent N, Walsh K. Web- vs. telehealth-based delivery of cognitive behavioral therapy for insomnia: a randomized controlled trial. Sleep Medicine. 2014 Feb;15(2):187-95. PMID: 24461370. doi: 10.1016/j.sleep.2013.10.013.	No ipCBTI condition

20	Jarnefelt H, Harma M, Martimo KP, Paajanen T, Paunio T, Sallinen M, et al. Non-pharmacological interventions of insomnia among shift workers: an RCT trial in occupational health setting. Sleep medicine. 2017;40:e147. PMID: CN-01463267.	Conference abstract or similar
21	Jarnefelt H, Harma M, Sallinen M, Paajanen T, Virkkala J, Martimo KP, Hublin C. Insomnia interventions among shift workers: an RCT trial in occupational health services. Sleep Science. 2019;12:13. PMID: CN-02049682.	Conference abstract or similar
22	Kim JE, Kim SS. The Effects of Mobile Social Networking Service-Based Cognitive Behavior Therapy on Insomnia in Nurses. Journal of Korean Academy of Nursing. 2017 Aug;47(4):476-87. PMID: 28894070. doi: 10.4040/jkan.2017.47.4.476.	Other language than English
23	Kim JI, Yun J-Y, Park H, Park S-Y, Ahn Y, Lee H, et al. A mobile videoconference-based intervention on stress reduction and resilience enhancement in employees: Randomized controlled trial. Journal of Medical Internet Research. 2018 Oct 22;20(10):12. PMID: 2174043893; 2018-57916-001. doi: https://doi.org/10.2196/10760.	Not CBTI
24	Kjørstad K, Sivertsen B, Vedaa Ø, Langsrud K, Faaland PM, Vethe D, et al. The Effect of Reducing Insomnia Severity on Work- and Activity-Related Impairment. Behavioal Sleep Medicine. 2021 Jul-Aug;19(4):505-15. PMID: 32731764. doi: 10.1080/15402002.2020.1799792.	Secondary analysis (and no outcomes of interest)
25	Lancee J, Effting M, van der Zweerde T, van Daal L, van Straten A, Kamphuis JH. Cognitive processes mediate the effects of insomnia treatment: evidence from a randomized wait-list controlled trial. Sleep Medicine. 2019 Feb;54:86-93. PMID: 30529782. doi: 10.1016/j.sleep.2018.09.029.	Secondary analysis (and no outcomes of interest)
26	Morawetz D. Behavioral self-help treatment for insomnia: A controlled evaluation. Behavior Therapy. 1989 1989/06/01/;20(3):365-79. doi: 10.1016/S0005-7894(89)80056-5.	Insufficient data
27	Okajima I, Akitomi J, Kajiyama I, Ishii M, Murakami H, Yamaguchi M. Effects of a Tailored Brief Behavioral Therapy Application on Insomnia Severity and Social Disabilities Among Workers With Insomnia in Japan: A Randomized Clinical Trial. JAMA Network Open. 2020 Apr 1;3(4):e202775. PMID: 32286659. doi: 10.1001/jamanetworkopen.2020.2775.	No ipCBTI condition
28	Peter L, Reindl R, Zauter S, Hillemacher T, Richter K. Effectiveness of an Online CBT-I Intervention and a Face-to-Face Treatment for Shift Work Sleep Disorder: A Comparison of Sleep Diary Data. International Journal of Environmental Research and Public Health. 2019 Aug 24;16(17). PMID: 31450619. doi: 10.3390/ijerph16173081.	Non-randomized allocation
29	Savard J, Ivers H, Savard MH, Morin C. Efficacy of a video-based psychological intervention for cancer-related insomnia: results of a randomized controlled trial. Journal of clinical oncology. 2014;32(15). PMID: CN-01009788.	Conference abstract or similar
30	Savard J, Ivers H, Savard MH, Morin CM, Caplette-Gingras A, Bouchard S, Lacroix G. Efficacy of a stepped care approach to deliver cognitive-behavioral therapy for insomnia in cancer patients: a noninferiority randomized controlled trial. Sleep. 2021 Nov 12;44(11). PMID: 34228123. doi: 10.1093/sleep/zsab166.	Non-randomized allocation

31	Savard J, Ivers H, Savard MH, Villa J, Morin C. Is a video-based cognitive-behavioral therapy as efficacious as a professionally administered treatment for insomnia comorbid with cancer? preliminary results of a randomized controlled trial. Psycho-oncology. 2013;22:15. PMID: CN-01006830. doi: 10.10002/pon.3245.	Conference abstract or similar
32	Savard J, Ivers H, Morin CM, Lacroix G. Video cognitive-behavioral therapy for insomnia in cancer patients: A cost-effective alternative. Psycho-Oncology. 2021 Jan;30(1):44-51. PMID: 2493135840; 2021-11315-001. doi: 10.1002/pon.5532.	Secondary analysis (and non-relevant outcomes)
33	Turkowitch D, Ludwig R, Nelson E, Drerup M, Siengsukon CF. Telehealth-Delivered Cognitive Behavioral Therapy for Insomnia in Individuals with Multiple Sclerosis: A Pilot Study. Multiple Sclerosis International. 2022 Mar 2;2022:7110582. PMID: 35281348. doi: 10.1155/2022/7110582.	Non-randomized allocation
34	Vincent N, Walsh K, Lewycky S. Sleep locus of control and computerized cognitive-behavioral therapy (cCBT). Behavior Research and Therapy. 2010 Aug;48(8):779-83. PMID: 20627268. doi: 10.1016/j.brat.2010.05.006.	Non-randomized allocation
35	Ye YY, Liu J, Li XJ, Liu YZ, Lang Y, Lin L, et al. The efficacy of internet-based cognitive behavioral therapy for insomnia. Medical journal of Chinese people's liberation army. 2016;41(4):307-11. PMID: CN-01265405. doi: 10.11855/j.issn.0577-7402.2016.04.09.	Other language than English

Table S3: Within-group pre-post effects of ipCBTI and eCBTI on primary sleep outcomes and secondary sleep and non-sleep outcomes

			ipCBTI			eCBTI		
**Time point**	**Outcome**	**K [a]**	**Effect (mean dif or Hedges's g[b])**	**95%CI**	***p* [c]**	**Effect (mean dif or Hedges's g[b])**	**95%CI**	***p* [c]**
**Post**	Total sleep disturb. (Hedges' g)	15	1.75	1.45; 2.06	**<.001**	1.28	1.06; 1.50	**<.001**
	ISI (mean difference, points)	11	-9.0	-10.3; -7.7	**<.001**	-7.1	-8.3; -5.9	**<.001**
	ISI (Hedges' g)	11	1.97	1.64; 2.31	**<.001**	1.36	1.12; 1.59	**<.001**
	PSQI (mean difference, points)	5	-4.4	-5.3; -3.5	**<.001**	-3.5	-4.7; -2.4	**<.001**
	PSQI (Hedges' g)	5	1.21	0.88; 1.54	**<.001**	0.99	0.61; 1.37	**<.001**
	Sleep efficiency, diary (mean difference, %)	11	12.1	10.0; 14.3	**<.001**	10.3	8.0; 12.7	**<.001**
	Sleep efficiency, diary (Hedges' g)	11	1.01	0.84; 1.19	**<.001**	0.92	0.73; 1.11	**<.001**
	SOL diary (mean difference, min)	10	-20.9	-24.8; -17.0	**<.001**	-19.6	-25.6; -13.5	**<.001**
	SOL diary (Hedges' g)	10	0.72	0.55; 0.90	**<.001**	0.65	0.49; 0.82	**<.001**
	WASO diary (mean difference, min)	8	-23.5	-31.6; -15.4	**<.001**	-19.5	-28.7; -10.3	**<.001**
	WASO (Hedges' g)	8	0.68	0.48; 0.89	**<.001**	0.57	0.35; 0.78	**<.001**
	TST (mean difference, min)	11	21.3	12.2; 30.4	**<.001**	16.3	5.2; 27.4	**.015**
	TST (Hedges' g)	11	0.27	0.16; 0.37	**<.001**	0.23	0.08; 0.39	**.003**
**FU**	Total sleep disturb. (Hedges' g)	14	1.73	1.42; 2.05	**<.001**	1.41	1.15; 1.68	**<.001**
	ISI (mean difference, points)	10	-8.5	-9.9; -7.2	**<.001**	-7.1	-8.3; -5.9	**<.001**

	ISI (Hedges' g)	10	1.88	1.52; 2.23	**<.001**	1.39	1.11; 1.66	**<.001**
	PSQI (mean difference, points)	5	-4.5	-5.8; -3.1	**<.001**	-3.8	-5.5; -2.0	**<.001**
	PSQI (Hedges' g)	5	1.11	0.69; 1.53	**<.001**	1.09	0.51; 1.67	**<.001**
	Sleep efficiency, diary (mean difference, %)	10	11.8	9.8; 13.8	**<.001**	9.2	6.3; 12.0	**<.001**
	Sleep efficiency, diary (Hedges' g)	10	0.97	0.84; 1.09	**<.001**	0.80	0.53; 1.06	**<.001**
	SOL diary (mean difference, min)	9	-20.1	-23.3; 17.0	**<.001**	-17.7	-24.4; -11.0	**<.001**
	SOL diary (Hedges' g)	9	0.71	0.57; 0.84	**<.001**	0.57	0.39; 0.75	**<.001**
	WASO diary (mean difference, min)	7	-18.0	-25.7; -10.3	**<.001**	-19.7	-29.4; -10.0	**.002**
	WASO (Hedges' g)	7	0.56	0.39; 0.72	**<.001**	0.57	0.36; 0.79	**<.001**
	TST (mean difference, min)	9	34.1	24.6; 43.7	**<.001**	28.8	17.1; 40.5	**<.001**
	TST (Hedges' g)	9	0.43	0.32; 0.53	**<.001**	0.39	0.22; 0.56	**<.001**
	**Actigraphy-based outcomes**							
**Post**	Sleep efficiency, actigraphy (mean difference, %)	3	2.03	-1.52; 5.58	.263	2.68	-1.22; 6.59	.178
	Sleep efficiency, actigraphy (Hedges' g)	3	0.27	-0.22-0.77	.278	0.32	-0.24; 1.07	.214
	SOL actigraphy (mean difference, min)	3	-11.53	-20.42; 2.64	**.011**	-8.86	-3.05; 20.77	.145
	SOL actigraphy (Hedges' g)	3	0.53	0.23; 0.83	**.001**	0.40	-0.16; 0.95	.162
	TST actigraphy (mean difference, min)	3	-27.48	-66.02; 11.06	.162	12.82	-16.34; 41.98	.389
	TST actigraphy (Hedges' g)	3	-0.47	-1.11; 0.18	.154	-0.24	-0.74; 0.28	.364
	**Secondary non-sleep outcomes**							
**Post**	Fatigue (Hedges' g)	6	0.74	0.57; 0.92	**<.001**	0.72	0.57; 0.87	**<.001**
	Anxiety (Hedges' g)	9	0.48	0.29; 0.66	**<.001**	0.51	0.34; 0.69	**<.001**

Depression (Hedges' g)	11	0.69	0.46; 0.92	**<.001**	0.67	0.43; 0.91	**<.001**

Abbreviations and explanations: ipCBTI: In-person delivered cognitive-behavioral therapy for insomnia; eCBTI: electronically-delivered (eHealth) CBTI; Post: post-intervention; FU: Follow-up; Sleep disturbance (All): combined self-report sleep outcomes, e.g., ISI, PSQI, HSDQ; ISI: Insomnia Severity Index; PSQI: Pittsburgh Sleep Quality Index; HSDQ: Holland Sleep Disorder Questionnaire; SE: Sleep Efficiency (%) ((TST/TiB)×100); SOL: Sleep Onset Latency (minutes); WASO: Wake after sleep onset (minutes); TST: Total sleep time (minutes); TiB: Time in bed; Diary: Sleep parameters based on sleep diaries, e.g., The Consensus Sleep Diary [1]; Actigr.: Sleep parameters based on actigraphy

Notes: a) K = number of studies; b) Analyses were conducted for outcomes with $K \geq 3$ for mean differences (%, minutes) and SMD (Hedges's g: Standardized Mean Difference adjusted for small sample bias) [2], with positive values of Hedges's g indicating difference of effects in the hypothesized direction, e.g. reduced insomnia severity and increased TST; c) *P*-values (two-tailed): Statistically significant ($p < 0.05$) in **bold**.

Table S4: Secondary non-sleep outcomes: Fatigue, anxiety, and depression

**Comparison: IpCBTI vs eCBT**				**Heterogeneity**		**Pooled effect**				**Equivalence [f]**		
**Time point**	**Self-reported outcomes**	**K [a]**	**N [b]**	***I²***	***T²***	**SMD [c] (Hedges' g)**	**95%CI**	***P* [d]**	**95%PI [e]**	**90%CI**	***p***	**MID**
**Post**	Fatigue	6	508	35.1	0.03	0.12	-0.11-0.34	.307	-0.44-0.67	-0.07-0.30	**<.001**	**0.50 [g]**
	Anxiety	9	700	37.3	0.03	0.03	-0.16-0.22	.768	-0.45-0.50	-0.13-0.19	**<.001**	**0.50 [g]**
	Depression	11	791	32.9	0.03	0.09	-0.09-0.26	.345	-0.34-0.51	-0.06-0.23	**<.033**	**0.25 [h]**
**FU**	Fatigue	6	508	00.0	0.00	0.11	-0.07-0.28	.279	N/A	-0.04-0.25	**<.001**	**0.50 [g]**
	Anxiety	9	700	13.0	0.01	-0.05	-0.21-0.11	.520	-0.34-0.23	-0,19-0.08	**<.001**	**0.50 [g]**
	Depression	11	791	16.3	0.01	0.05	-0.11-0.21	.520	-0.25-0.35	-0.08-0.18	**.006**	**0.25 [h]**

Abbreviations and explanations: IpCBTI: In-person delivered cognitive-behavioral therapy for insomnia; eCBTI: electronically-delivered (eHealth) CBTI; Post: post-intervention; FU: Follow-up; MID = Minimal Important Difference (or Clinical Significance Threshold) (see e.g., [3].

Notes: a) K = number of studies; b) N= total number of participants; c) Analyses were conducted for outcomes with K ≥ 3 for SMDs (Hedges's g: Standardized Mean Difference adjusted for small sample bias) [2], with positive values indicating difference of effects in favor of IpCBTI; d) P-values (two-tailed): Statistically significant ($p < 0.05$) in **bold**. e) 95% prediction interval, i.e., the interval in which 95% of future observations from the same family of studies will fall [4]; f) Test of equivalence: tests whether the confidence interval (CI) falls within an equivalence interval. The equivalence test is based on the largest *p*-value from two one-sided tests [5]. P-values marked with ***** indicate equivalence. The tests are based on the following minimal important differences (MIDs) of: g) A SD of 0.50 as suggested by [6], when no MIDs were available, and h) a SD of 0.25, based on the MID suggested by [7]. All but one comparison (Fatigue, FU) remained statistically significantly equivalent when using 0.25 SD as the equivalence margin.

Table S5: Results of supplementary Bayesian meta-analyses of the post-intervention results

		Equivalent (H_1) vs. non-zero difference between IpCBTI and eCBTI (H_0)				Non-heterogeneity (H_0) vs. heterogeneity (H_1)			
	K	Favors H_1	Favors H_0	BF	Evidence level	Favors H_0	Favors H_1	BF	Evidence level
Insomnia severity (ISI)	11	-	X	10.5	Strong	-	X	13.0	Strong
Sleep quality (PSQI)	5	X	-	1.7	Anecdotal	-	X	2.3	Anecdotal
Sleep efficiency	11	-	X	1.2	Anecdotal	X	-	2.2	Anecdotal
Sleep onset latency (SOL)	10	X	-	8.2	Moderate	X	-	1.9	Anecdotal
Wake after sleep onset (WASO)	8	X	-	6.0	Moderate	X	-	2.0	Anecdotal
Total Sleep Time (TST)	11	X	-	9.9	Moderate	X	-	3.5	Moderate

Abbreviations and explanations: ipCBTI: In-person delivered cognitive-behavioral therapy for insomnia; eCBTI: electronically-delivered (eHealth) CBTI; Bayes Factor interpretation, adapted from [8]: 1-3 = Anecdotal; 3-10 = Moderate; 10-30 = Strong; 30-100 = Very strong; >100 = Decisive

Figure S1: Risk of Bias (RoB 2)

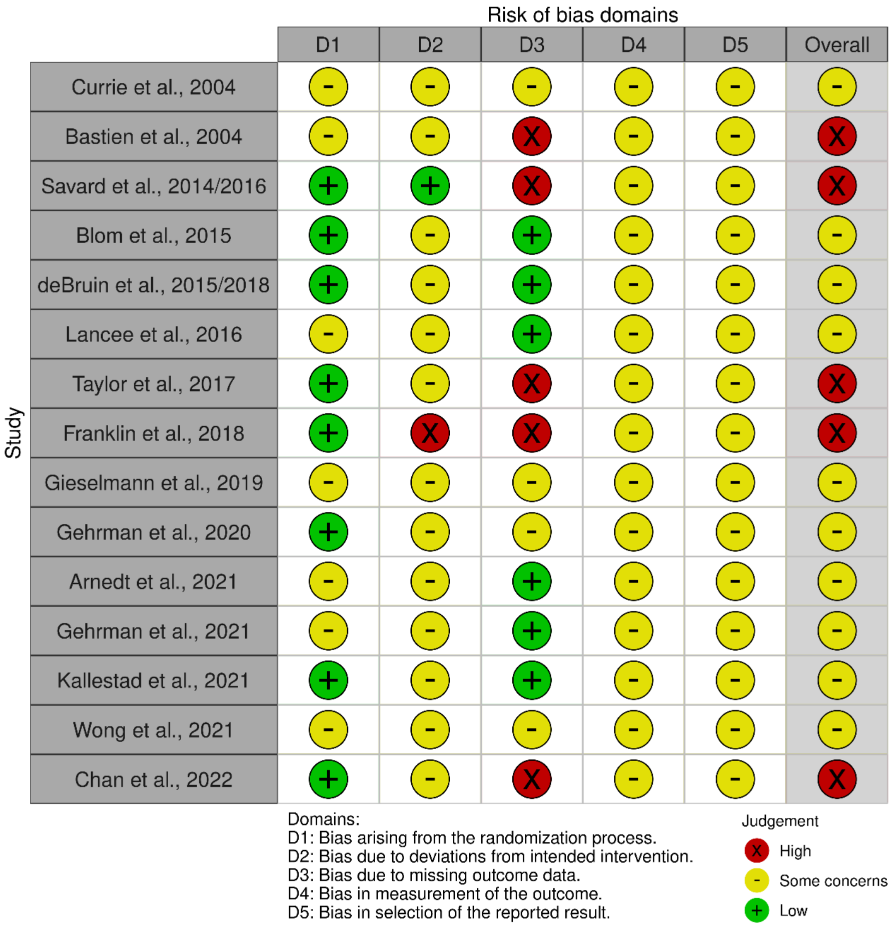

Summary plot [9]

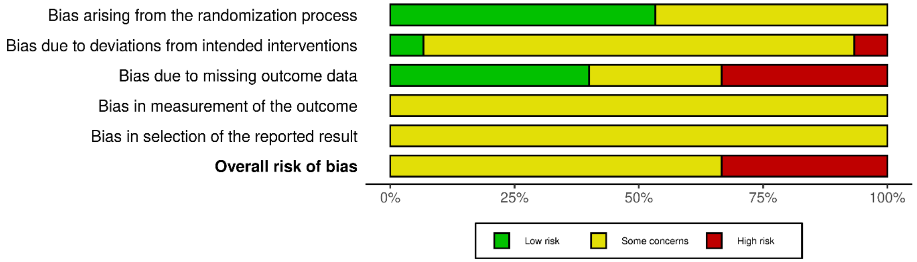

Figure S2: Forest plot of post-intervention mean differences (points) between effects of eCBTI and ipCBTI on insomnia severity (ISI)

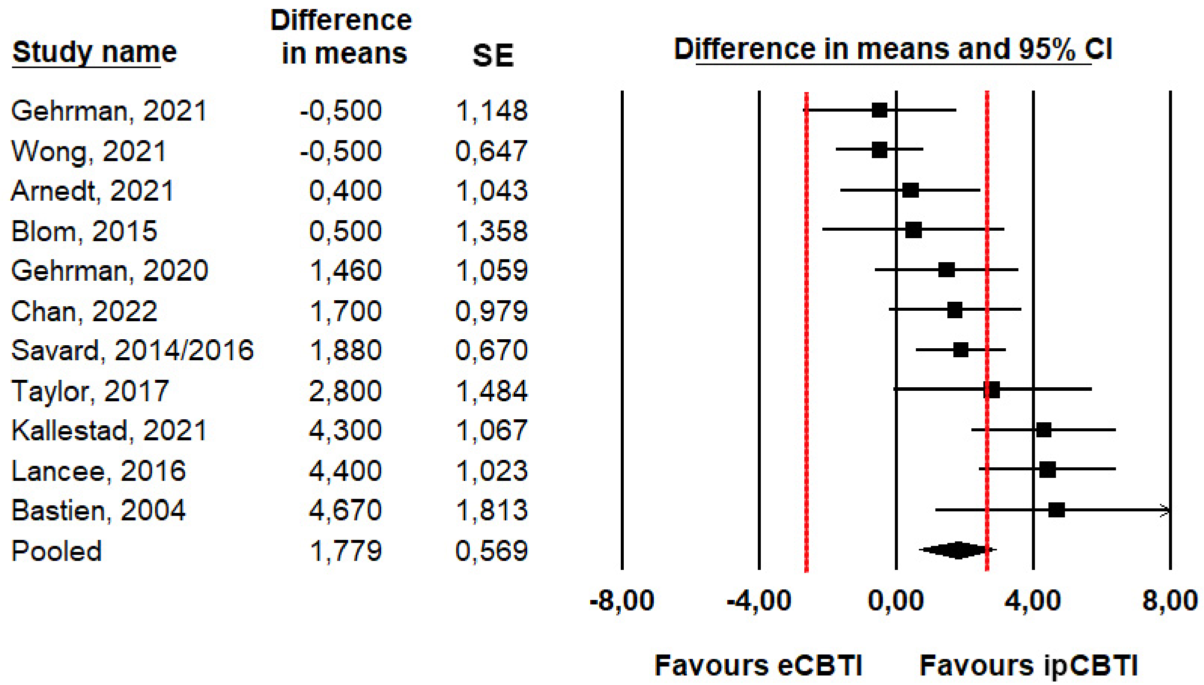

Abbreviations and explanations: eCBTI: electronically-delivered (eHealth) CBTI; ipCBTI: In-person delivered cognitive-behavioral therapy for insomnia; red lines represent the equivalence margin based on a minimal important difference (MID) of 2.6 points, corresponding to 0.5 SD [3, 10]

Figure S3: Forest plot of post-intervention differences (Hedges's g) between effects of eCBTI and ipCBTI on insomnia severity (ISI)

Study name	g	SE
Gehrman, 2021	-0,133	0,307
Wong, 2021	-0,130	0,168
Arnedt, 2021	0,094	0,245
Blom, 2015	0,105	0,284
Gehrman, 2020	0,281	0,205
Chan, 2022	0,363	0,211
Savard, 2014/2016	0,440	0,159
Taylor, 2017	0,456	0,245
Kallestad, 2021	0,796	0,205
Bastien, 2004	0,930	0,381
Lancee, 2016	1,096	0,274
Pooled	0,371	0,115

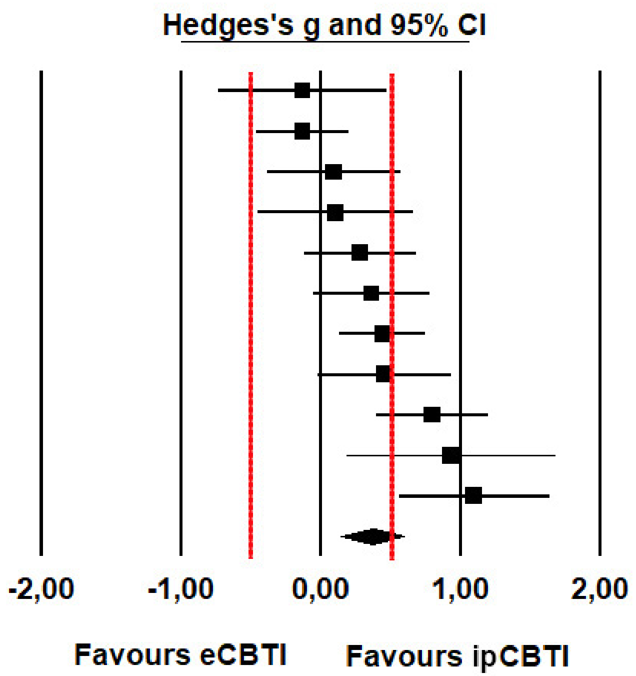

Abbreviations and explanations: eCBTI: electronically-delivered (eHealth) CBTI; ipCBTI: In-person delivered cognitive-behavioral therapy for insomnia; red lines represent the equivalence margin based on the suggested minimal important difference (MID) of 0.5 SD [3]

Figure S4: Forest plot of post-intervention mean differences (points) between effects of eCBTI and ipCBTI on sleep quality (PSQI)

Study name	Difference in means	SE
Gieselmann, 2019	-1,480	0,914
Gehrman, 2020	0,550	0,796
Chan, 2022	1,200	0,545
Franklin, 2018	2,300	2,313
Currie 2004	3,300	1,325
Pooled	0,859	0,720

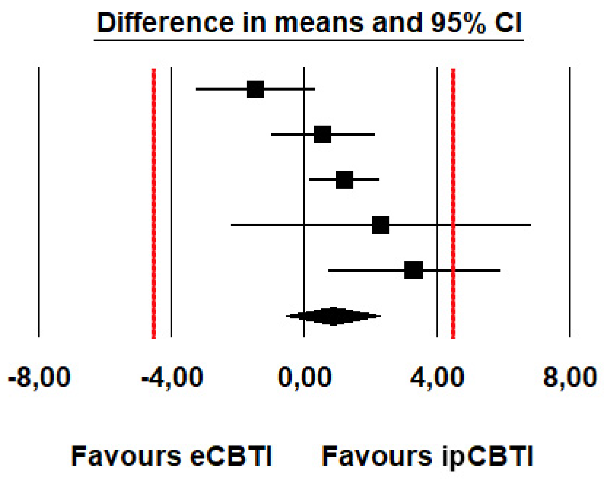

Abbreviations and explanations: eCBTI: electronically-delivered (eHealth) CBTI; ipCBTI: In-person delivered cognitive-behavioral therapy for insomnia; red lines represent the equivalence margin based on the suggested minimal important difference (MID) of 4.4. points [11].

Figure S5: Forest plot of post-intervention differences (Hedges's g) between effects of eCBTI and ipCBTI on sleep quality (PSQI)

Study name	g	SE
Gieselmann, 2019	-0,452	0,283
Franklin, 2018	0,515	0,527
Gehrman, 2020	0,141	0,204
Chan, 2022	0,460	0,212
Currie 2004	0,871	0,367
Pooled	0,259	0,209

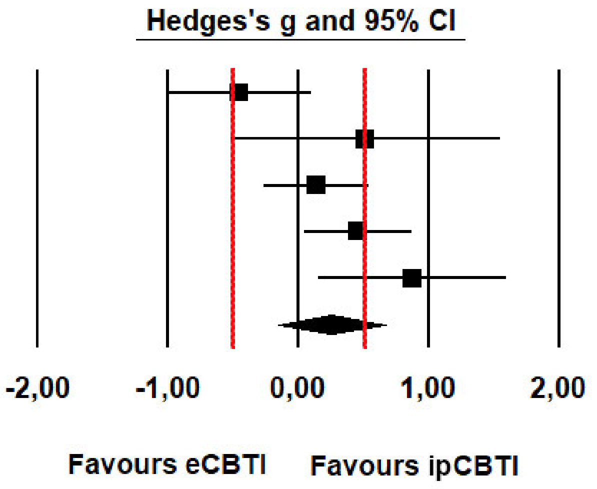

Abbreviations and explanations: eCBTI: electronically-delivered (eHealth) CBTI; ipCBTI: In-person delivered cognitive-behavioral therapy for insomnia; red lines represent the equivalence margin based on the suggested minimal important difference (MID) of 0.5 SD [3].

Figure S6: Forest plot of post-intervention differences (Hedges's g) between effects of eCBTI and ipCBTI on diary-based sleep efficiency

Study name	g	SE
Kallestad, 2021	-0,102	0,198
Arnedt, 2021	-0,051	0,245
Bastien, 2004	-0,010	0,361
deBruin 2015/2018	0,027	0,226
Gieselmann, 2019	0,100	0,279
Lancee, 2016	0,259	0,256
Savard, 2014/2016	0,260	0,158
Currie 2004	0,325	0,352
Taylor, 2017	0,337	0,243
Chan, 2022	0,350	0,211
Blom, 2015	0,443	0,288
Pooled	0,174	0,071

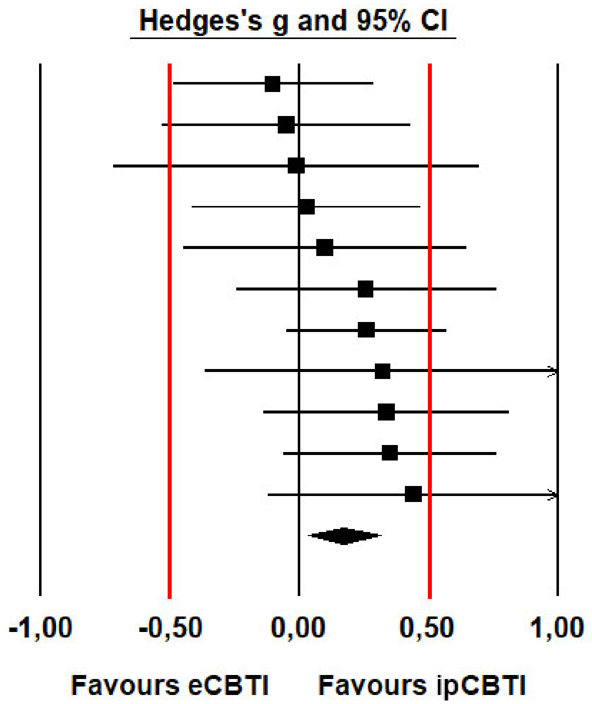

Abbreviations and explanations: eCBTI: electronically-delivered (eHealth) CBTI; ipCBTI: In-person delivered cognitive-behavioral therapy for insomnia; red lines represent the equivalence margin based on the suggested minimal important difference (MID) of 0.5 SD [3].

Figure S7: Forest plot of post-intervention mean differences (minutes) between effects of eCBTI and ipCBTI on diary-based sleep onset latency (SOL)

Study name	Difference in means	SE
Bastien, 2004	-10,420	11,877
Blom, 2015	-6,000	9,687
Gieselmann, 2019	-4,710	7,827
Kallestad, 2021	-1,400	7,811
deBruin 2015/2018	-1,000	9,280
Currie 2004	-0,300	9,656
Arnedt, 2021	0,500	6,795
Savard, 2014/2016	2,270	3,218
Taylor, 2017	4,000	6,703
Chan, 2022	12,800	4,555
Pooled	2,614	1,956

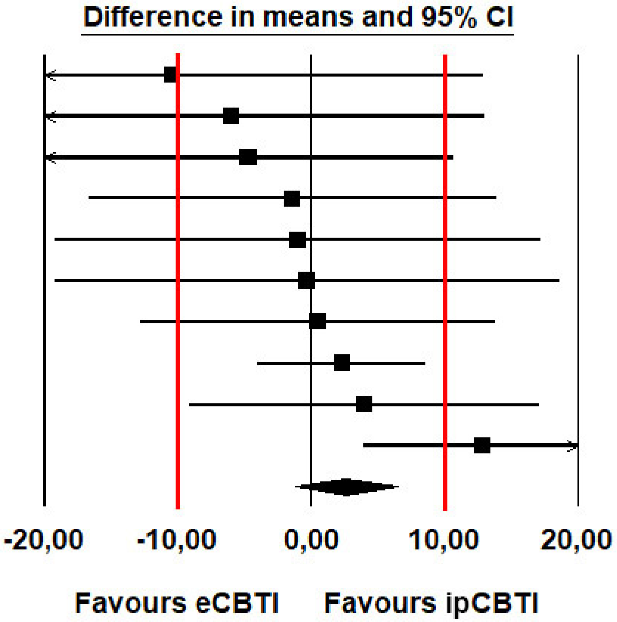

Abbreviations and explanations: eCBTI: electronically-delivered (eHealth) CBTI; ipCBTI: In-person delivered cognitive-behavioral therapy for insomnia; red lines represent the equivalence margin based on the suggested minimal important difference (MID) of 10 minutes [3]

Figure S8: Forest plot of post-intervention differences (Hedges's g) between effects of eCBTI and ipCBTI on diary-based sleep onset latency (SOL)

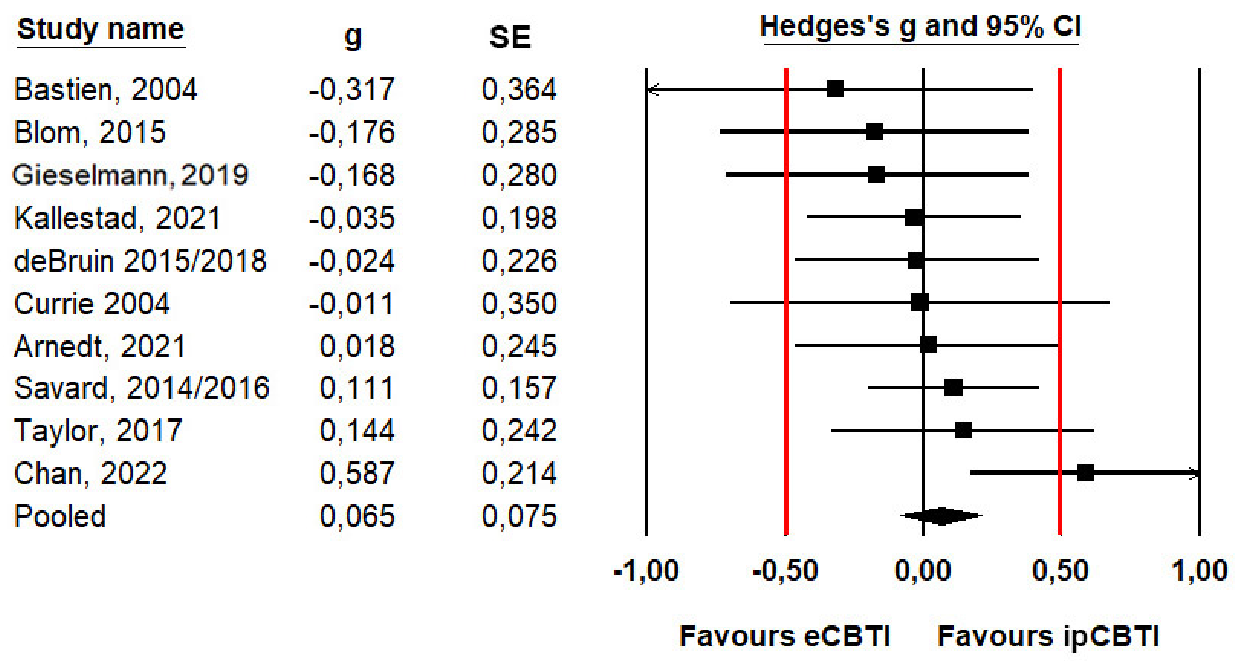

Abbreviations and explanations: eCBTI: electronically-delivered (eHealth) CBTI; ipCBTI: In-person delivered cognitive-behavioral therapy for insomnia; red lines represent the equivalence margin based on a suggested minimal important difference (MID) of 0.5 [6]

Figure S9: Forest plot of post-intervention mean differences (minutes) between effects of eCBTI and ipCBTI on diary-based wake after sleep onset (WASO)

Study name	Difference in means	SE
Arnedt, 2021	-14,000	10,505
Kallestad, 2021	-4,800	7,487
Chan, 2022	-0,600	4,363
Savard, 2014/2016	3,860	3,661
deBruin 2015/2018	5,000	5,803
Taylor, 2017	13,000	8,913
Currie 2004	18,000	18,347
Bastien, 2004	25,910	17,178
Pooled	2,484	2,579

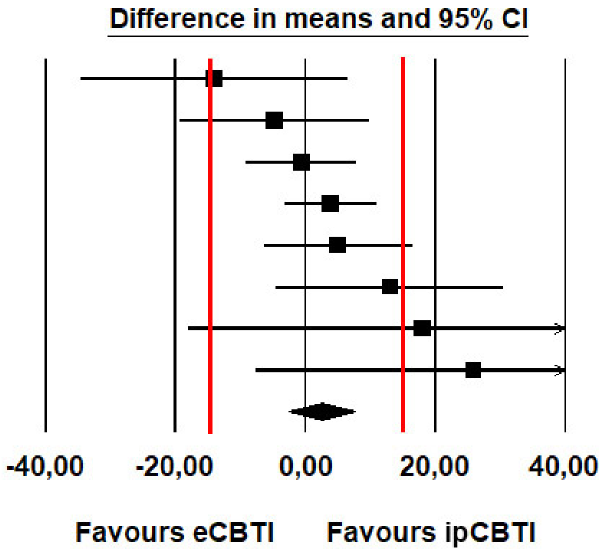

Abbreviations and explanations: eCBTI: electronically-delivered (eHealth) CBTI; ipCBTI: In-person delivered cognitive-behavioral therapy for insomnia; red lines represent the equivalence margin based on the suggested minimal important difference (MID) of 15 minutes [3].

Figure S10: Forest plot of post-intervention differences (Hedges's g) between effects of eCBTI and ipCBTI on diary-based wake after sleep onset (WASO)

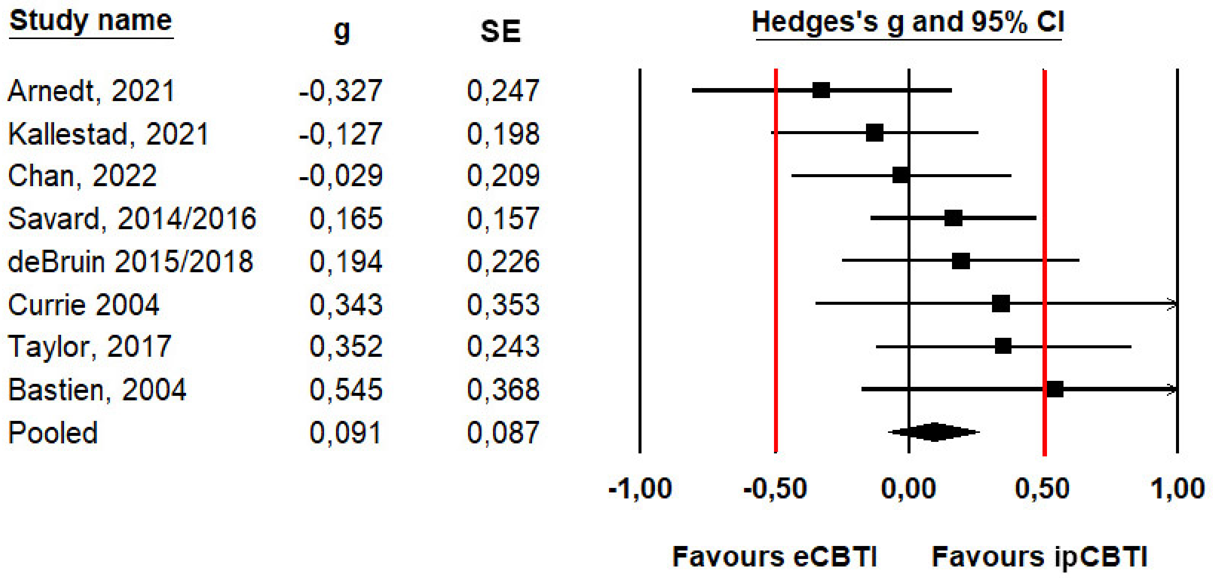

Abbreviations and explanations: eCBTI: electronically-delivered (eHealth) CBTI; ipCBTI: In-person delivered cognitive-behavioral therapy for insomnia; red lines represent the equivalence margin based on a suggested minimal important difference (MID) of 0.5 SD [6].

Figure S11: Forest plot of post-intervention differences (Hedges's g) between effects of eCBTI and ipCBTI on diary-based total sleep time (TST)

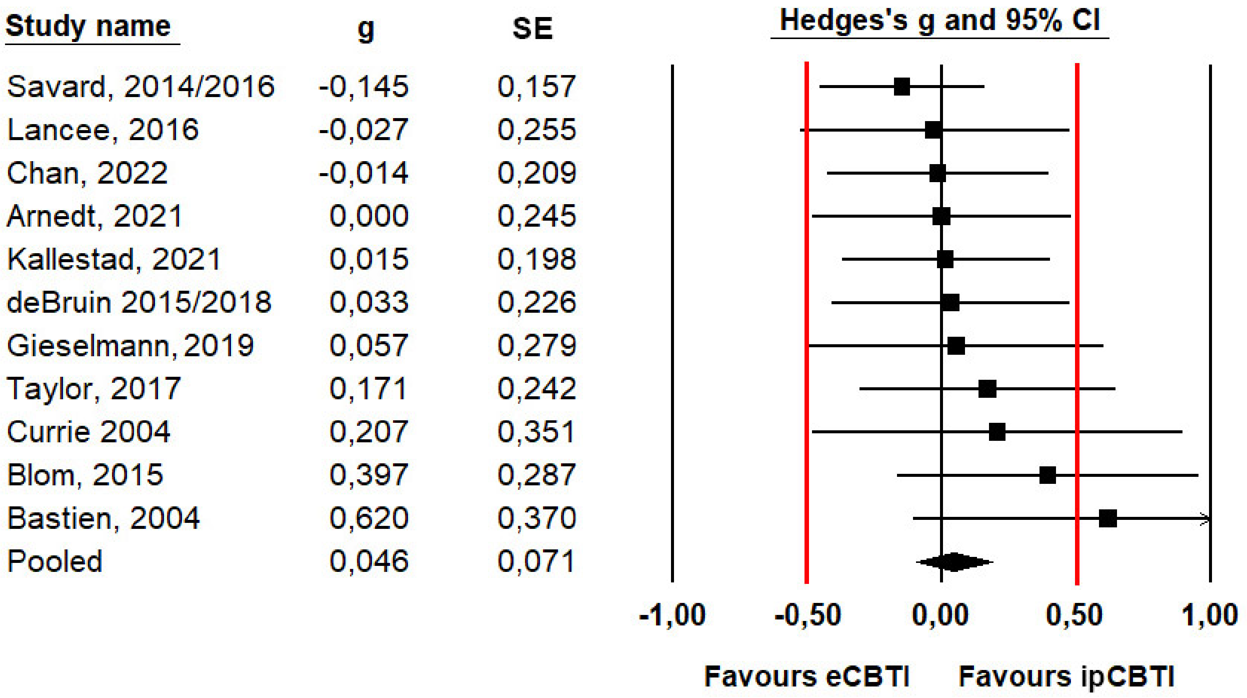

Abbreviations and explanations: eCBTI: electronically-delivered (eHealth) CBTI; ipCBTI: In-person delivered cognitive-behavioral therapy for insomnia; red lines represent the equivalence margin based on a suggested minimal important difference (MID) of 0.5 SD [6].

Figure S12: Funnel plot of post-intervention differences (Hedges's g) between effects of eCBTI and ipCBTI on total sleep disturbance

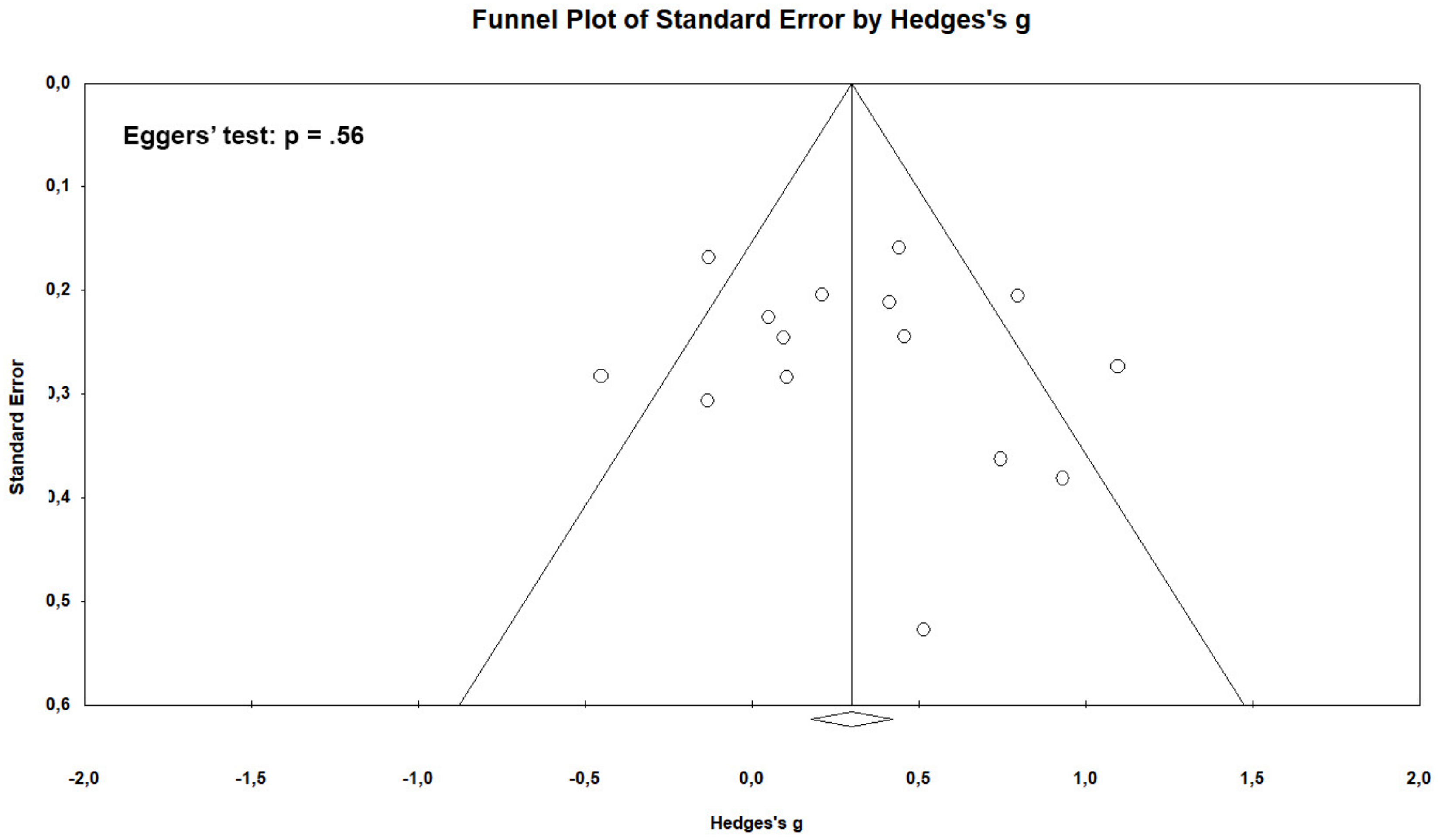

References to figures and tables

1. Carney CE, Buysse DJ, Ancoli-Israel S, Edinger JD, Krystal AD, Lichstein KL, et al. The consensus sleep diary: standardizing prospective sleep self-monitoring. Sleep. 2012 2/1/2012;35(2):287-302.

2. Hedges L, Olkin I. Statistical methods for meta-analysis. New York: Academic Press; 1985 1985.

3. Edinger JD, Arnedt JT, Bertisch SM, Carney CE, Harrington JJ, Lichstein KL, et al. Behavioral and psychological treatments for chronic insomnia disorder in adults: an American Academy of Sleep Medicine systematic review, meta-analysis, and GRADE assessment. J Clin Sleep Med. 2021 Feb 1;17(2):263-98. PMID: 33164741. doi: 10.5664/jcsm.8988.

4. IntHout J, Ioannidis JP, Rovers MM, Goeman JJ. Plea for routinely presenting prediction intervals in meta-analysis. BMJ Open. 2016 Jul 12;6(7):e010247. PMID: 27406637. doi: 10.1136/bmjopen-2015-010247.

5. Rogers JL, Howard KI, Vessey JT. Using significance tests to evaluate equivalence between two experimental groups. Psychol Bull. 1993 May;113(3):553-65. PMID: 8316613. doi: 10.1037/0033-2909.113.3.553.

6. Norman GR, Sloan JA, Wyrwich KW. Interpretation of changes in health-related quality of life: the remarkable universality of half a standard deviation. MedCare. 2003 5/2003;41(5):582-92. doi: 10.1097/01.MLR.0000062554.74615.4C [doi].

7. Cuijpers P, Turner EH, Koole SL, van Dijke A, Smit F. What is the threshold for a clinically relevant effect? The case of major depressive disorders. Depress Anxiety. 2014 May;31(5):374-8. PMID: 24677535. doi: 10.1002/da.22249.

8. Jeffreys H. The theory of probability. 3 ed. Oxford, UK: Oxford University Press; 1961.

9. McGuinness LA, Higgins JPT. Risk-of-bias VISualization (robvis): An R package and Shiny web app for visualizing risk-of-bias assessments. Res Synth Methods. 2021 Jan;12(1):55-61. PMID: 32336025. doi: 10.1002/jrsm.1411.

10. Bastien CH, Vallieres A, Morin CM. Validation of the Insomnia Severity Index as an outcome measure for insomnia research. Sleep Med. 2001 7/2001;2(4):297-307. doi: S1389945700000654 [pii].

11. Longo UG, Berton A, De Salvatore S, Piergentili I, Casciani E, Faldetta A, et al. Minimal Clinically Important Difference and Patient Acceptable Symptom State for the Pittsburgh Sleep Quality Index in Patients Who Underwent Rotator Cuff Tear Repair. Int J Environ Res Public Health. 2021 Aug 17;18(16). PMID: 34444415. doi: 10.3390/ijerph18168666.
